# Cross-tissue Analysis of Gene and Protein Expression in Normal and Cancer Tissues

**DOI:** 10.1038/srep24799

**Published:** 2016-05-04

**Authors:** Idit Kosti, Nishant Jain, Dvir Aran, Atul J. Butte, Marina Sirota

**Affiliations:** 1 Institute for Computational Health Sciences, University of California, San Francisco, California, United States of America

## Abstract

The central dogma of molecular biology describes the translation of genetic information from mRNA to protein, but does not specify the quantitation or timing of this process across the genome. We have analyzed protein and gene expression in a diverse set of human tissues. To study concordance and discordance of gene and protein expression, we integrated mass spectrometry data from the Human Proteome Map project and RNA-Seq measurements from the Genotype-Tissue Expression project. We analyzed 16,561 genes and the corresponding proteins in 14 tissue types across nearly 200 samples. A comprehensive tissue- and gene-specific analysis revealed that across the 14 tissues, correlation between mRNA and protein expression was positive and ranged from 0.36 to 0.5. We also identified 1,012 genes whose RNA and protein expression was correlated across all the tissues and examined genes and proteins that were concordantly and discordantly expressed for each tissue of interest. We extended our analysis to look for genes and proteins that were differentially correlated in cancer compared to normal tissues, showing higher levels of correlation in normal tissues. Finally, we explored the implications of these findings in the context of biomarker and drug target discovery.

In recent years, techniques used to conduct tissue-wide analysis of gene expression, such as microarrays and RNA sequencing technologies (RNA-Seq), have become widely used^1^,^2^. An example of an effort in this area is the Genotype-Tissue Expression (GTEx) project, which contains RNA-Seq measurements from 43 different tissues in hundreds of samples^3^. As central functional units in many complex biological pathways, proteins are also a subject of much interest in various areas of translational medicine, including diagnostic biomarker discovery, drug discovery and personalized medicine. Measuring global protein levels directly in human tissue samples, however, has traditionally presented many challenges, as well as major difficulties including reproducibility^4^. Recent advances in protein analysis technology provide methods for exploring the relationship between mRNA expression and protein abundance. For example, mass spectrometry (MS)^5^ has been used to map the proteomes of yeasts, worms, and flies^6^. MS-based comparative analysis of several human cell lines has also been conducted^7^. Other technologies, such as immunohistochemistry, produce images that aid in measuring levels of protein expression^8^. However, these methods do not scale to capture genome-wide protein measurements. Thus, despite advances in high-throughput methods for proteomics, the relationship between gene expression and protein abundance is still unclear.

Comparative studies have found that correlations between mRNA and protein levels in model organisms can be relatively weak and uncertain or moderately positive^9^, and that they vary between experiments and organisms. For example, Gygi *et al.* observed a moderately positive Pearson correlation of R = 0.48 when studying a subset of proteins in *S. cerevisiae*. Correlations vary greatly among genes, depending on regulatory processes that govern the rates of translation and protein degradation^10^. Schwanhausser *et al.* obtained similar results in mouse fibroblasts, where expression of a subset of 5000 genes was moderately correlated with protein levels (R = 0.44)^11^. In humans, Gry *et al.* described a similar lower relationship in 23 cell lines, where R values ranged from 0.25 to 0.52, depending on the methodology that was applied^12^. Studies of gene and protein expression correlation in cancer tissues are less common, and findings are often contradictory. For example, in bone osteosarcoma, squamous cell carcinoma and brain glioblastoma, concordance has been shown to be high (R values ranging from 0.58 and 0.63)^7^, while in lung adenocarcinomas, R values ranged from −0.467 to 0.442^13^. In the aforementioned projects, sample sizes were small and analysis focused on a subset of genes.

To date, a comprehensive study of the correlation between gene expression and protein abundance in the human body has yet to be performed. With the release of two independent MS-based draft maps of the human proteome in May 2014, researchers now have unprecedented access to a database of system-wide tissue-specific protein levels^14^,^15^. One of these efforts, the Human Proteome Map Project (HPM), cataloged proteins corresponding to 84% of the known protein-encoding genes in the human genome across 30 different human tissues^14^. Combined with the existing transcriptomic data libraries from GTEx, these seminal advances have created an opportunity to explore the central dogma of biology and document correlations between gene expression and protein levels.

Here, we present a large-scale analysis of protein abundance and gene expression across a diverse set of human tissues. We examined ~80% of human genes for tissue- and gene-specific correlations. Furthermore, we extend our initial analysis to determine which genes and tissues are correlated in their protein abundance and gene expression in cancer tissues to inform the identification of new drug targets and biomarkers.

## Results

We analyzed the GTEx and HPM datasets and found that 16,561 genes and their corresponding proteins were represented in both repositories across 14 tissues. More than 39,000 transcripts, consisting mainly of non-coding RNAs, were present only in the GTEx dataset, while 688 proteins were present only in the HPM data (Supplementary Fig. S1). These data were excluded from our analysis. The number of genes expressed in each tissue in this combined dataset varied from 9,937 (heart) to 13,054 (testis). The number of proteins present per tissue varied from 5,022 (esophagus) to 11,030 (testis; Supplementary Table S1).

### Correlating Gene and Protein Expression Across Tissues

**Figure 1:**
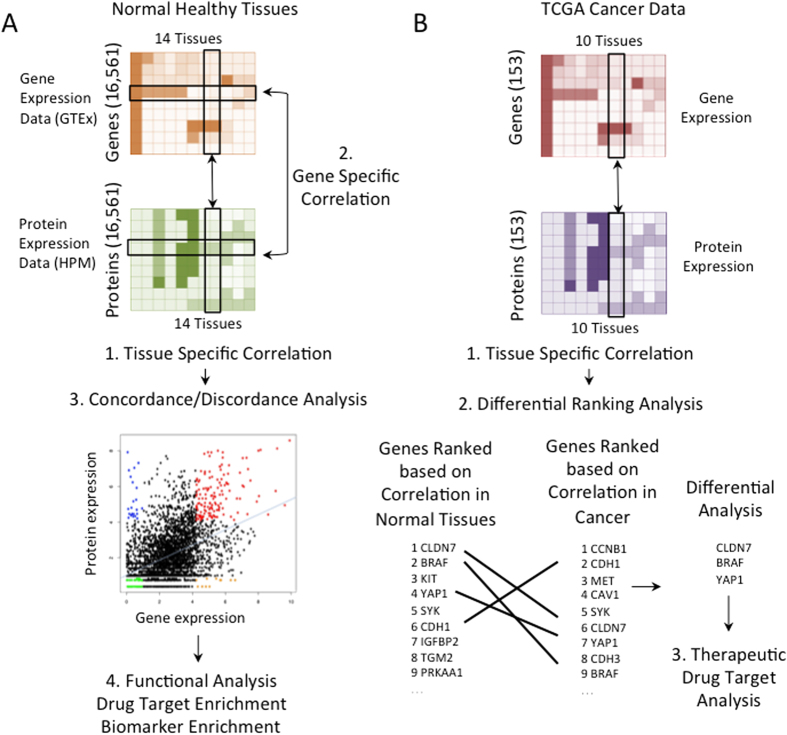
Analysis overview. (**A**) Analysis of normal tissues based on GTEx and HPM expression data. (1) Tissue-specific correlations (2) Gene-specific correlations (3) Concordance/discordance analysis (4) Functional analysis including drug targets and biomarkers. (**B**) Cancer and normal tissues analysis based on TCGA data. (1) Tissue-specific correlation (2) Differential ranking analysis (3) Therapeutic drug target analysis.

**Figure 2:**
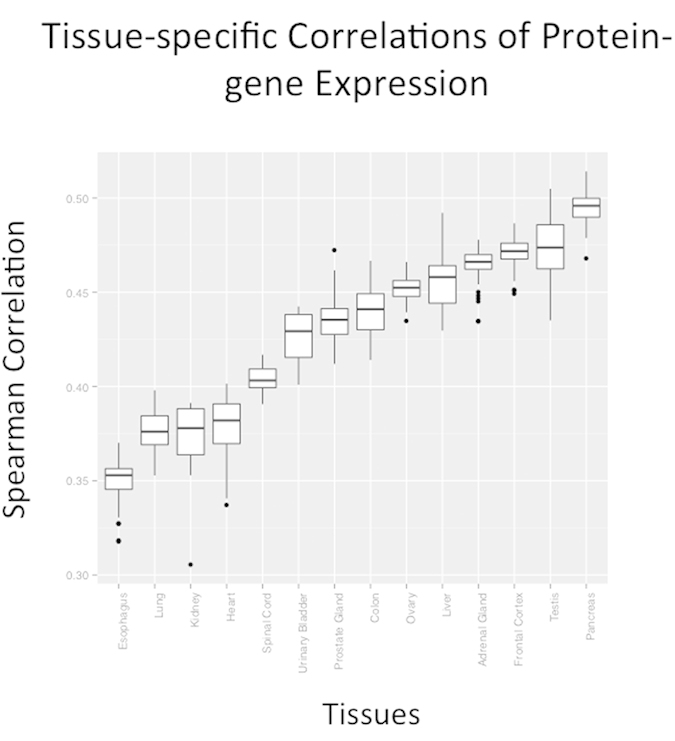
Tissue-specific correlations of protein-gene expression. Tissues are ranked by their average Spearman correlation value.

For each tissue, GTEx transcriptomic samples were paired with a corresponding proteomic measurement from the HPM dataset. A Spearman correlation was calculated for each pair. Fig. 1A illustrates this analysis (comparison 1), while Fig. 2 shows the distributions of correlations per tissue. Correlations ranged from 0.36 to 0.50, with a median of 0.45. Esophageal tissue had the lowest score distribution and pancreatic tissue had the highest (supplementary Fig. S2). These results agree with previous attempts to correlate mRNA expression with protein abundance^10^,^11^,^12^.

**Figure 3:**
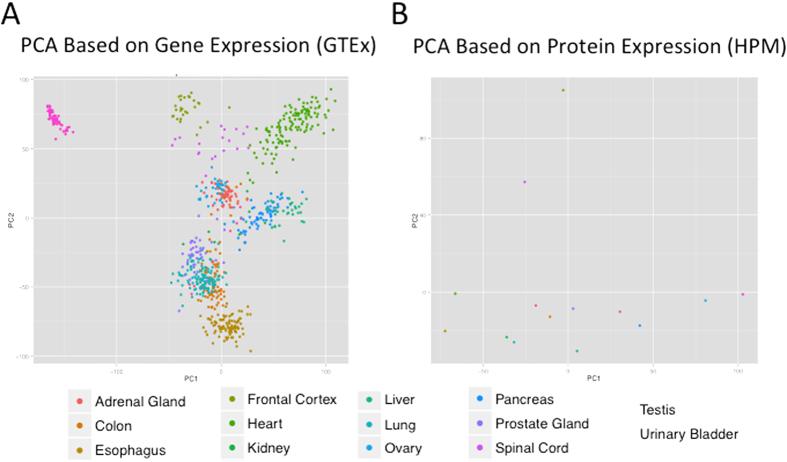
Tissue-specific correlations of protein-gene expression. (**A**) PCA based on gene expression. (**B**) PCA based on protein expression.

We compared tissue similarity by performing principal component analysis (PCA) and by comparing the topology of hierarchical clusters based on either GTEx or HPM data (Fig. 3). In both PCA plots, there were similarities between different tissue types, with prostate, colon, and urinary bladder samples clustering together in both gene and protein expression analysis. Testis, frontal cortex, and spinal cord samples were outliers. PCA revealed a high similarity between GTEx samples from the same tissues (also shown previously^16^), which led us to use medians for all relevant samples per tissue in the next step of the analysis.

We found a matching clustering pattern through hierarchical clustering approaches (Supplementary Fig. S4A,B) based on gene and protein expression. The gene expression dendrogram in Fig. S4A shows three main clusters with p*-*values  < 0.05 (marked in red). They include the following-cluster one: ovary, colon, urinary bladder, prostate; cluster two: lung, kidney; cluster three: spinal cord, testis. The HPM dendrogram in Supplementary Fig. S4B shows four main groups with significant correlations for all tree branches (p-value < 0.05, significant clusters marked in red): cluster one: heart, esophagus; cluster two: frontal cortex, spinal cord; cluster three: prostate, colon, urinary bladder; and cluster four: liver, adrenal gland, kidney, ovary, testis, lung, pancreas. To measure similarity between the two trees, we used cophenetic correlation based on Spearman correlations. The analysis returned a weak value (c = 0.25), illustrating the different gene and protein expression landscapes. The only significant similarity between the dendrograms was the clustering of the prostate gland, colon and urinary bladder tissues.

**Figure 4:**
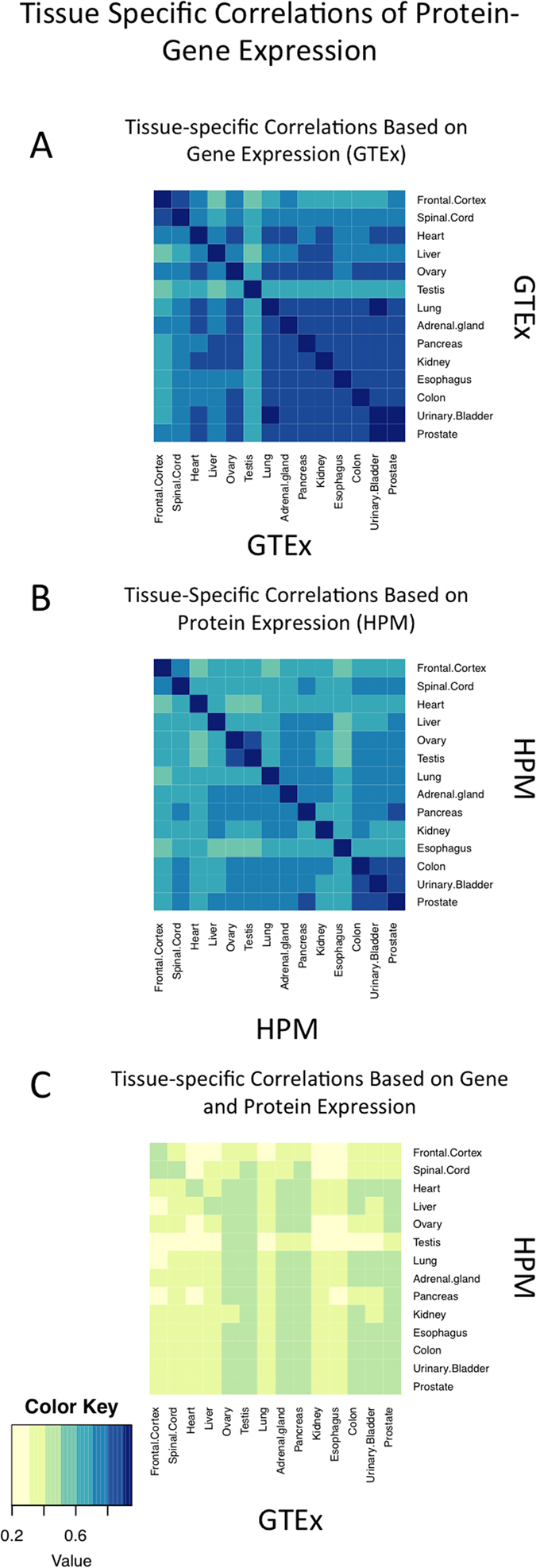
(**A**) Tissue-specific correlations of gene expression (GTEx). (**B**) Tissue-specific correlations of protein expression (HPM). (**C**) Tissue-specific correlations of protein-gene expression.

We used sample similarities to examine the relationships of gene and protein expression between different tissues. We first correlated all tissues based on gene expression (Fig. 4A) and protein expression (Fig. 4B) using the Spearman correlation metric (see Supplementary Fig. S2 for data distributions). The tissues with the highest correlation based on gene expression were observed on the diagonal of the matrix, which was expected. For many tissues in the GTEx dataset, gene expression correlation values were very similar among tissues forming a large cluster (rho values: 0.81–1). Expression in the frontal cortex was very similar to that of the spinal cord (rho values: 0.85–1; Fig. 4A). Gene expression correlation in testicular samples was low with all tissues except itself.

Highly correlated protein expression among tissues was once again on the diagonal of the matrix. We saw high concordance in the following tissues: colon, urinary bladder, and prostate (rho values: 0.83–1), spinal cord and frontal cortex (rho values: 0.765–1), and ovary and testis (rho values: 0.87–1; Fig. 4B).

Finally, we correlated the tissues based on their protein abundance and gene expression across all tissues analyzed (Fig. 4C). Overall correlations were lower than gene-gene correlations (highest values), and protein-protein comparisons. In many tissues, we found that a gene-protein pairing in a single tissue had one of the highest correlation values; however, we found many other tissue pairings in which one tissue’s gene expression profile was correlated more strongly with protein expression from another tissue, suggesting similarities between tissues (Fig. 4C for median correlation values and Supplementary Fig. S3 for sample pairwise correlation values). An example is the frontal cortex and spinal cord. Both are part of the central nervous system, and both share similar gene and protein expression profiles. In other cases, such as testis, connections between gene and protein expression were asymmetric. A gene-protein correlation heatmap (Fig. 4C) shows that while GTEx testis gene expression had similarities to many tissues in HPM, the HPM protein expression of the testis only seemed to correlate with the GTEx testis and ovary samples.

### Identifying significantly correlated genes and proteins across all tissues

In order to identify genes that were highly correlated with protein expression across all tissues, we computed Spearman correlations for each gene, correlating mRNA and protein expression across all 14 tissues. Figure 1A has an illustration of the analysis (comparison 2). Overall, statistically significant correlation between mRNA expression and protein abundance was observed in only 1,012 genes out of 16,561 (6.1%). Rho values ranged from 0.77 to 1 (p-value <0.05; Supplementary Fig. S2). Of these genes, 262 (~25%) were expressed in only one tissue. They were predominantly in the testis (~47%) and the frontal cortex (~21%), both of which are known for unique expression patterns^17^,^18^. The remaining 750 genes showed a significant correlation between mRNA expression and protein abundance across at least two tissues (Supplementary Table S2). Furthermore, there was correlation across all tissues in 169 out of 1,012 genes (17%).

In this group of genes and proteins with highly correlated expression, we found representatives of different cellular classes, such as the splicing factor SRSF6, the demethylase KDM5A, the ribosome-binding protein of the endoplasmic reticulum RRBP1, and the immune system regulator HLA-A. The genes in these examples share basic essential cellular functions. GO annotation analysis revealed a high enrichment of genes involved in oxidation reduction (Benjamini adjusted p-value: 9.3E-09), and various transport activities, including ion transport (Benjamini adjusted p-value: 2.6E-05) and transmembrane transporter activity (Benjamini adjusted p-value: 1.3E-04), both performing basic functions of the cell.

This highly correlated group of genes and proteins is of special interest because it contains genes and proteins that are known biomarkers and drug targets. We carried out functional analysis of highly correlated genes and the full gene set by using Ingenuity Pathway Analysis (IPA). We identified 101 genes known to be biomarkers in our 1,012 highly correlated genes set (Table 1), comprising 10% of the highly correlated genes. When the same analysis was performed on the full gene set, only 6.1% of genes were identified as biomarkers. This comparison shows enrichment for biomarker genes in the highly correlated gene set (p-value: 8.1E-06, chi-square test). One example is *CKB*, Creatine Kinase chain B (rho: 0.84, p-value: 2.8E-04). *CKB* is a biomarker used in drug safety experiments and a suggested biomarker for cardiovascular diseases^19^ and squamous cell lung cancer^20^. It is quantified in serum for accurate measurement by different proteomic methods.

We also studied known drug targets in the full and highly correlated gene sets. The highly correlated set was enriched for drug targets (142 genes out of 1,012, p-value: 0.02, chi-square test) compared to the full set (1,551 genes out of 16,551). The 142 highly correlated genes are targets for 449 different drugs according to the DrugBank^21^ (Table 2). Drug targets include genes such as Protein Kinase C, Alpha (*PRKCA*, rho: 0.77, p-value: 0.04) which is targeted by tamoxifen, and Gamma-Aminobutyric Acid A Receptor, Alpha 3 (*GABRA3*, rho; 1, p-value: < 2.2E-16) which is targeted by diazepam.

### Tissue-specific concordance-discordance analysis

In the full dataset, we identified a set of 983 genes, which were not expressed in any of the tissues, but for which protein expression was observed. These genes were highly enriched in sensory perception annotations (see Supplementary Table S3 for full annotations list with p-values). We also identified 1,200 proteins which were not expressed in our dataset, but corresponding mRNA expression was observed. These genes were also highly enriched in the regulation of transcription (see Supplementary Table S4 for full annotations list with p-values).

We further characterized several groups of interest with respect to gene and protein expression by tissue. We analyzed four “corner case” groups: high gene expression-high protein expression, high gene expression-low protein expression, low gene expression-high protein expression, and low gene expression-low protein expression (see Materials and Methods and Supplementary Figs S2 and S5). We examined genes whose over- or under-expression was in the top 10% in a given tissue, and found that, on average, 240 genes fell into the high gene expression-high protein expression group, 20 fell into the low gene expression-high protein expression group, 25 fell into the high gene expression-low protein expression group and 138 fell into the low gene expression-low protein expression group (Supplementary Figs S2 and S5). The functions of genes in these groups are well-characterized^11^. For example, genes whose expression was low tend to have longer 3′ UTRs with AU rich elements and specific TF binding sites.

We studied known drug targets in the context of these four gene sets (Supplementary Fig. S6, Supplementary Tables S5–S8). Most targets had high and concordant gene-protein expression (70–91% across tissues), showing that expression of target genes as measured with microarrays corresponded well to protein levels in the treated tissue. This finding was also true for genes with low and concordant gene-protein expression, the second biggest group (8–19% across tissues). Targets with high gene expression-low protein expression and low gene expression-high protein expression were poorly represented (0–4% and 0–11% across tissues, respectively). These two discordant groups should get close attention, as therapeutic decisions based on gene expression can lead to critical mistakes.

### Identifying differentially correlated genes based on protein and gene expression in cancer

**Figure 5:**
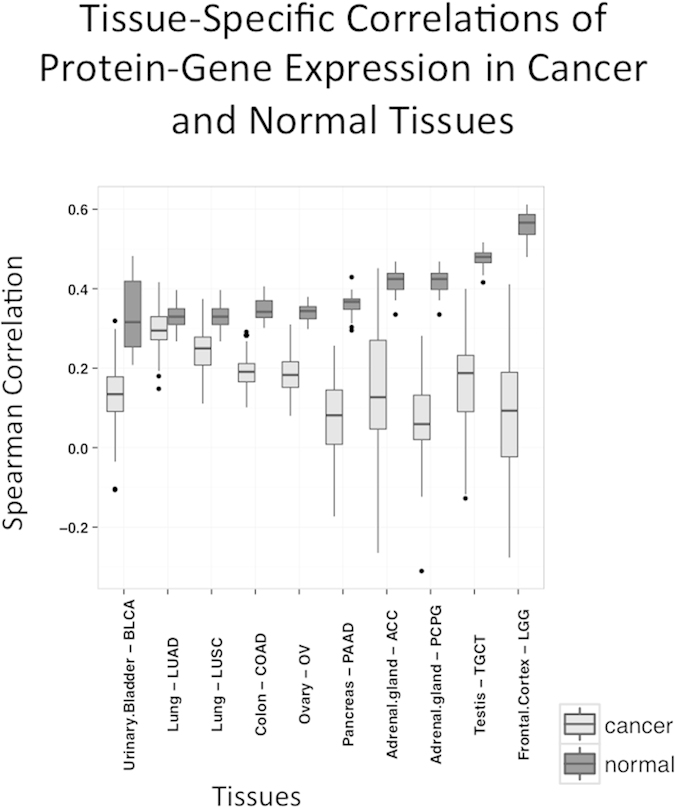
Tissue-specific correlations of protein-gene expression for 10 tumor types and corresponding normal tissues: adenoid cystic carcinoma (ACC—adrenal gland: pheochromocytoma and paraganglioma (PCPG)—adrenal gland; bladder urothelial carcinoma (BLCA)—urinary bladder; colon adenocarcinoma (COAD)—colon; lower grade glioma (LGG)—frontal cortex; lung adenocarcinoma (LUAD)—lung; lung squamous cell carcinoma (LUSC)—lung; ovarian serous cystadenocarcinoma (OV)—ovary; pancreatic adenocarcinoma (PAAD)—pancreas.

We studied differential gene-protein correlation in cancers, using mRNA and protein data from the Cancer Proteome Atlas (TCPA). TCPA is a cancer functional proteomics database and is a part of the Cancer Genome Atlas (TCGA) project^22^. Like our analysis for normal tissues, we compared the Spearman correlation between mRNA and protein expression patterns for 153 genes in 10 types of cancer from TCPA with 8 corresponding normal tissues (adrenal gland, urinary bladder, colon, frontal cortex, lung, ovary, pancreas, and testis) from the GTEx and HPM datasets (Fig. 1B, marked as comparison number 1). Figure 5 shows our results. Interestingly, Spearman RNA-protein correlations for this set of genes were lower in almost all cancer types compared to corresponding normal tissues. The correlation was similar in only one cancer (lung adenocarcinoma, LUAD).

To further explore differences between cancer and normal tissues, we investigated changes in the relationship between gene and protein expression by gene, in tissue pairs. For each cancer-normal tissue pair, we compared the ranking of genes based on the correlation of gene and protein expression. Table 3 summarizes genes that showed differential behavior between cancer and normal tissues (See Supplementary Tables S9 and S10 for full data on genes that were correlated in cancer). One highly correlated gene in cancer vs. normal was Y box binding 1 (*YBX1*), which was differentially correlated in ACC, COAD, LUAD and LUSC. *YBX1* is a transcription factor and a splicing factor. It controls many genes involved with cancer, such as p53^23^. It is known for overexpression in cancer, and is involved in malignant progression of colorectal adenocarcinoma^24^,^25^ and lung cancer^26^, among other tumors. It is also associated with poor survival. A recent paper suggests that tRNA-derived fragments can suppress breast cancer progression via binding to *YBX1*, and by that mechanism, prevent it from binding pro-oncogenic transcripts^27^. RAC-alpha serine/threonine-protein kinase (*AKT1*) is another gene that was highly correlated in cancer (LGG, PAAD and TGCT). *AKT1* is a target in cancer therapy^28^ via several drugs^29^. There was a strong correlation between its mRNA and protein levels in some of the cancer types in this study, but not in normal tissues. Because it has three known isoforms^30^, we hypothesize that targeting *AKT1* at the mRNA level and identifying a cancer-specific isoform may lead to better chemotherapies.

Finally, we compared the genes with differential gene-protein expression relationships to drug targets in the DrugBank database^21^. Table 4 lists all drug targets that were differentially correlated between cancer and normal tissues. An example of a gene with highly correlated protein expression in cancer is receptor tyrosine-protein kinase erbB-2 (*ERBB2*), which is targeted in LUAD and LUSC that by ado-trastuzumab emtansine, afatinib, and trastuzumab. Another example is Proto-oncogene Tyrosine-protein Kinase (*SRC*), which is targeted by bosutinib, dasatinib, and ponatinib in COAD. Gene expression measurements in both of these cases and others can be used (or are already in use) as biomarkers and drug targets. Other genes on our list showing differential protein-gene expression correlation in cancer have the potential to be used as new biomarkers and drug targets.

## Discussion

The publication of datasets such as the Human Proteome Map (HPM)^14^ enables researchers to ask new questions regarding proteins in different tissues. Although it is clear that the number of detected proteins does not resemble the full scope of the protein landscape, due to alternative splicing and other similar processes^28^, the HPM dataset provides an informative glimpse into the human proteome. Our first goal in the current analysis was to explore connections between mRNA levels and protein abundances, in a large-scale study, using publically available data in normal human tissues. We achieved this goal by combining proteomic data from the HPM project and mRNA expression data from the GTEx project.

Our analysis revealed a positive gene-protein expression correlation (0.36–0.5) for the majority of human tissues. For a subset of genes, there was a statistically significant relationship between mRNA expression and protein abundance in all measured tissues. Furthermore, for each tissue we identified a set of genes and proteins that were concordantly expressed at high and low levels, and a much smaller set of genes and proteins that were discordantly expressed. Surprisingly, for genes that were highly correlated across all tissues, or highly concordantly expressed in individual tissues, we could not find an overall strong functional enrichment within the group. This result suggested that a diverse set of genes is under similar regulatory pressure that shapes their correlation. Furthermore, we identified two interesting groups, those with gene expression but no protein expression, and vice versa. Non-detectable expression may be due to technical limitations, a short half-life of the mRNA or protein, or, in the case of RNA expression only, non-coding RNAs. Surprisingly, non-detectable levels of gene expression had no effect on the levels of the detected protein expression, suggesting fast translation in the case of a short half-life or efficient translation from a small amount of mRNA in the case of a low level of mRNA.

We performed functional analysis of gene sets that are known biomarkers and drug targets. A bottleneck in the usage of biomarkers is the long and expensive process required for proteomic measuring of each sample. Measuring mRNA expression levels is cheaper, but is insufficient to determine protein levels because correlations between mRNA expression and protein abundance are relatively low. We found that gene-protein expression of biomarkers and drug targets was more correlated than expected across all tissues and in tissue-specific analysis. These biomarkers are already in use for diagnosis, prognosis, to monitor disease progression, drug efficacy, drug safety and/or response to treatment. mRNA expression and protein abundance was highly correlated in these biomarkers, implying that mRNA expression levels may, in some cases, be sufficient to determine protein abundance. This approach would allow for easier estimates of protein levels.

Although the highly correlated biomarkers and drug targets were the vast majority, we found that gene and protein expression was uncorrelated for some biomarkers and drug targets. These genes are important for further study and for consideration in the context of drug discovery or therapeutic decisions. Gene expression measurements that do not correspond to protein measurements could lead to inaccurate therapeutic decisions. Clearly, many processes can affect drug efficacy, such as gene and protein expression for drug transport^29^.

In order to investigate the relationships of gene and protein expression in disease, we used TCPA, a cancer functional proteomics dataset that is a part of the TCGA project. By choosing genes based on their differential correlation of mRNA and protein expression, we were able to identify a set of such genes across all cancer types and within each cancer tissue that contains a number of known biomarkers and therapeutic targets. This list can be used in combination with the traditional differential expression analysis for new biomarker and drug target discovery. We hypothesize that testing a patient for highly correlated mRNA transcript before treating him with a drug against the corresponding protein could improve treatment outcomes by improving treatment target specificity.

Our study has several limitations that should be recognized. The first is the source of our data. Unfortunately, the GTEx and HPM data were not collected in a single experiment on the same samples. This fact may affect measurements of expression and correlation across the two experiments. To overcome this limitation, we used the Spearman correlation metric in our tissue-specific and gene-specific analysis. This approach is based on rank rather than on expression values. Another limitation is our ability to work with available protein data. Recently, Protein Atlas, another source of protein expression based on antibody staining to determine protein expression, was published^8^. Due to collection methodology, protein expression data in that resource is categorical and cannot be used in the same way as the continuous HPM data. For this reason, it was omitted from our analysis. Finally, our cancer analysis examined only 153 genes. These genes were carefully chosen for the study. Therefore, it is not surprising to find a connection between them and cancer. As more untargeted proteomics-based normal and tumor datasets are generated in the next several years, the approaches presented here can be applied more extensively.

In conclusion, we have performed a systematic analysis to examine the central dogma of biology and look for similarities and differences between protein and gene expression in a large number of healthy and cancer tissues. We found that different tissues are differently correlated in their protein and gene expression. We also found that gene and protein expression was highly correlated across tissues in a small set of genes. We further identified a set of genes that were concordantly abundant in each tissue based, on gene and protein levels. This analysis found that a statistically significant proportion of these genes are known biomarkers and therapeutic targets. We also examined genes with discordant gene and protein expressions levels, and hypothesize that those with opposite behavior should be under proper consideration if being detected only by gene expression levels. We finally extended our analysis to diseased tissues, focusing on cancer and identified lower gene protein concordance in cancer in comparison to normal tissues. We posit that concordance and discordance in gene and protein expression has important implications for therapeutics and diagnostics.

## Materials and Methods

### Dataset sources

The mRNA data in this analysis was extracted from the Genotype-Tissue Expression (GTEx) Project (http://www.GTExportal.org), which is based on RNA-seq data expression levels in various human tissue samples^3^. The GTEx project contains data from a combination of sources and technologies, including gene expression microarrays and RNA sequencing. Of the 53 tissue types in the project, we chose 18 tissues corresponding to 14 HPM tissues. The final subset of the GTEx data included RNA sequencing of 921 tissue samples, each of which belonged to one of the 18 tissue subtypes, while esophagus, colon, and heart had more than one tissue. To remove bias, transcript reads on a gene-level basis were normalized for gene length, resulting in transcript data that was in the form of reads per kilobase per million (RPKM).

Protein data was extracted from the Human Proteome Map (HPM) Project (http://www.humanproteomemap.org). Data in this project is based on mass spectroscopy (MS) protein levels in various tissue samples across the systems of the human body^14^. As part of the HPM project, 30 histologically normal tissue types were profiled in totality, 17 of which were adult human tissues. For each tissue type, samples from three individuals were pooled and analyzed using MS. The project detected proteins corresponding to 17,294 genes, or ~84% of the protein-encoding regions of the human genome. In this work, 14 of the 17 adult tissues corresponding to tissue gene expression data existed in the GTEx database are in use. A subset of 16,571 genes that were commonly measured in HPM and GTEx datasets were used in this work.

Both datasets were evaluated in terms of data quality, detection limit, and number of samples per category. Expression values <1 in both datasets were counted as zero values.

For the cancer analysis, RNA-seq and reverse-phase protein arrays (RPPA) data from the Cancer Proteome Atlas (TCPA) was extracted for ten cancer types: adenoid cystic carcinoma (ACC), pheochromocytoma and paraganglioma (PCPG), bladder urothelial carcinoma (BCLA), colon adenocarcinoma (COAD), lower grade glioma (LGG), lung adenocarcinoma (LUAD), lung squamous cell carcinoma (LUSC), ovarian serous cystadenocarcinoma (OV), pancreatic adenocarcinoma (PAAD), and testicular germ cell tumors (TGCT). These cancers correspond to eight normal tissues: adrenal gland (ACC and PCPG), urine bladder (BCLA), colon (COAD), frontal cortex (LGG), lung (LUAD and LUSC), ovary (OV), pancreas (PAAD), and testis (TGCT). A total of 153 genes were compared between TCPA and GTEx-HPM data.

### Tissue correlation

The Spearman correlation was calculated between gene and protein measurements across all tissues. Correlation values are presented in a heatmap. Clustering of data was done by pvclust^30^, based on correlation as the distance method and Ward’s method as the hierarchical clustering method. Statistically significant clusters (p-value:  < 0.05) are marked in red. Dendrogram comparison using cophenetic correlation was performed using the R package dendextend http://cran.r-project.org/web/packages/dendextend.

### Identifying and analyzing highly correlated genes across and within different tissues

An adjusted p-value of the Spearman correlation was used on the full dataset to create a list of 1,012 highly correlated genes across all tissues. From this set, 750 genes with correlations of two or more tissues were chosen for further annotation.

### Functional analysis for highly correlated genes across and within different tissues

Functional annotation was performed for the group of highly correlated genes across all tissues, and for 14 groups of tissue-specific highly correlated genes, using several tools. The GO annotation Molecular function was conducted using DAVID^31^. REVIGO^32^ was used for summarizing GO annotation terms. Biomarker gene annotation was performed using IPA (www.ingenuity.com). A comparison with drug targets was performed using data from the DrugBank database^21^, while confining the search to drug targets.

### Concordant and discordant expression analysis in GTEx and HPM dataset

We divided the data into four groups by using a top and bottom 10% criteria for each tissue. A high gene expression-high protein expression group included all data points whose gene and protein expression were in the top 10%. The high gene expression-low protein expression group contained all data points in the top 10% in the gene expression dataset and those in the bottom 10% in the protein expression dataset. The low gene expression-high protein expression group included all data points in the bottom 10% for gene expression and the top 10%, while the low gene expression-low protein expression group contains data points in the bottom 10% in both gene and proteins expression datasets. Drug target analysis was performed as explained for highly correlated genes across all tissues.

### Characterization of correlation and differential ranking in the cancer dataset

Spearman correlation between protein and gene expression was calculated across all tissues and within each tissue. For ranking comparison, the GTEx-HPM dataset was reduced to the same 153 genes as the cancer dataset. We sorted both datasets based on Spearman’s rho and ranked the results. We then compared the ranking based on gene-protein expression in normal and cancer datasets. A gene was considered as differentially correlated if the difference between the ranking based on the cancer dataset and the ranking based on the normal expression set was at least 30.

### Functional annotation for cancer and normal data

Drug target analysis was performed as explained for highly correlated genes across all tissues only for genes showing differential correlation in at least one cancer tissue.

## Additional Information

**How to cite this article**: Kosti, I. *et al.* Cross-tissue Analysis of Gene and Protein Expression in Normal and Cancer Tissues. *Sci. Rep.* **6**, 24799; doi: 10.1038/srep24799 (2016).

## Supplementary Material

Supplementary Table 1

Supplementary Table 2

Supplementary Table 3

Supplementary Table 4

Supplementary Information

## Figures and Tables

**Table 1:** Highly correlated biomarkers, with correlation score and corrected p-value, and number of tissues used for correlation calculation.

Gene symbol	Correlation	P-value corrected	# tissues usedfor correlation
CRH	1	<2.2E-16	1
GCG	1	<2.2E-16	2
GRM1	1	<2.2E-16	1
IAPP	1	<2.2E-16	1
IDO1	1	<2.2E-16	1
INS	1	<2.2E-16	1
LINGO2	1	<2.2E-16	1
NKX2-5	1	<2.2E-16	1
OPCML	1	<2.2E-16	2
SLC22A1	1	<2.2E-16	1
SLC28A1	1	<2.2E-16	2
S100B	0.95	1.20E-05	9
ALDH1A1	0.94	<2.2E-16	14
HGD	0.94	2.30E-05	6
KCNMA1	0.94	4.40E-05	8
RBP1	0.92	<2.2E-16	13
ARSE	0.92	1.40E-04	4
AGR2	0.91	3.90E-04	8
KRT18	0.89	<2.2E-16	14
TPM2	0.89	<2.2E-16	14
MUC1	0.89	9.40E-04	7
PDE5A	0.88	1.50E-03	8
FLNC	0.88	1.70E-03	14
DAO	0.87	2.80E-03	4
CDH1	0.87	3.00E-03	12
CLDN3	0.87	3.10E-03	8
CD36	0.87	3.30E-03	12
LCN2	0.86	3.30E-03	13
CDH2	0.86	3.80E-03	14
CLDN1	0.86	3.80E-03	9
ABCC3	0.86	4.00E-03	12
HPGD	0.86	4.30E-03	7
SST	0.85	4.80E-03	3
GGT1	0.85	4.90E-03	11
FGF1	0.85	5.00E-03	7
ITGA3	0.85	5.80E-03	13
RTN4	0.85	5.80E-03	14
CLDN4	0.84	7.20E-03	7
ACTA2	0.84	8.60E-03	14
CKB	0.84	1.00E-02	14
CDH13	0.83	9.20E-03	14
CRYAB	0.83	1.40E-02	14
PDCD4	0.83	1.40E-02	14
SERPINB5	0.82	1.30E-02	6
GNA12	0.82	1.50E-02	14
S100A4	0.82	1.50E-02	14
ABCB1	0.81	1.50E-02	10
NCAM1	0.81	1.60E-02	13
PEMT	0.81	1.60E-02	6
GSTM3	0.81	2.10E-02	14
LAMA5	0.81	2.10E-02	14
FOLR1	0.8	1.80E-02	4
ALOX5	0.8	2.00E-02	11
CAT	0.8	2.50E-02	14
ANPEP	0.79	2.10E-02	13
DAPK1	0.79	2.10E-02	4
TPD52	0.79	2.20E-02	14
MMP7	0.79	2.40E-02	8
CHGA	0.79	2.50E-02	10
TAGLN	0.79	3.20E-02	14
KRT19	0.78	2.50E-02	14
BAK1	0.78	2.70E-02	10
SPP1	0.78	2.90E-02	4
LGALS3	0.78	3.50E-02	14
SOD2	0.78	3.50E-02	14
TFRC	0.78	3.70E-02	14
CEACAM6	0.77	3.00E-02	6
FOLH1	0.77	3.30E-02	7
HLA-A	0.77	4.20E-02	14
NOS2	0.76	3.60E-02	3
P2RX7	0.76	3.80E-02	6
KIF1A	0.76	3.90E-02	12
DHCR7	0.76	4.20E-02	14
MYLK	0.76	4.20E-02	14
FABP5	0.75	4.20E-02	14
KRT17	0.75	4.20E-02	14
SULT1A1	0.75	4.20E-02	14
APOE	0.75	4.40E-02	14
GPX2	0.74	4.20E-02	13
ITGB4	0.74	4.20E-02	12
WFS1	0.74	4.20E-02	12
EPHX1	0.74	4.90E-02	14
CACNG2	0.73	4.20E-02	2
CASR	0.73	4.20E-02	1
CDX1	0.73	4.20E-02	1
CDX2	0.73	4.20E-02	1
CYP1A1	0.73	4.20E-02	2
CYP24A1	0.73	4.20E-02	1
CYP3A4	0.73	4.20E-02	1
GPR158	0.73	4.20E-02	1
KLK13	0.73	4.20E-02	1
KLK2	0.73	4.20E-02	1
KLK8	0.73	4.20E-02	2
MAGEB10	0.73	4.20E-02	2
ANXA3	0.73	4.40E-02	14
FXYD3	0.73	4.50E-02	6
LGALS4	0.72	4.70E-02	7
SPARCL1	0.72	5.00E-02	12
CSF1	−0.73	4.60E-02	8

**Table 2:** Complete list of highly correlated drug targets across all tissues.

Gene symbol	Drug(s)
ABAT	L-Glutamic Acid,Phenelzine,Pyridoxal Phosphate,Pyruvic acid,Valproic Acid,Vigabatrin,Adenosine triphosphate
ABCB1	FM-VP4,Roxithromycin,NADH
ACADS	NADH
ACAT1	Sulfasalazine
ACSBG1	2-(6-HYDROXY-1,3-BENZOTHIAZOL-2-YL)-1,3-THIAZOL-4(5H)-ONE
ACY3	L-Aspartic Acid
ADCY5	2′,5′-DIDEOXY-ADENOSINE3′-MONOPHOSPHATE
ADH1B	Fomepizole,NADH,Glycine
AGXT2	L-Alanine,Pyridoxal Phosphate,Pyruvic acid,3,4-DIHYDRO-4-OXO-3-((5-TRIFLUOROMETHYL-2-BENZOTHIAZOLYL)METHYL)-1-PHTHALAZINE ACETIC ACID
AKR1B10	3,4-DIHYDRO-4-OXO-3-((5-TRIFLUOROMETHYL-2-BENZOTHIAZOLYL)METHYL)-1-PHTHALAZINE ACETIC ACID
ALDH1A1	Tretinoin,Vitamin A,NADH
ALDH1B1	NADH
ALDH6A1	NADH
ALOX5	Balsalazide,Diclofenac,Diethylcarbamazine,Masoprocol,Meclofenamic acid,Mesalazine,Minocycline,MLN-977,Montelukast,Sulfasalazine,Vitamin E,Zileuton,Ezetimibe
ANPEP	Icatibant,Hydralazine
AOC3	Phenelzine,Human Serum Albumin
APOE	Serum albumin iodonated,Phosphatidylserine
ATP8A1	Phosphatidylserine
BAAT	Glycine
BHMT	L-Methionine
CA12	Ellagic Acid,Hydrochlorothiazide,Hydroflumethiazide,Zonisamide,Acetazolamide
CA4	Bendroflumethiazide,Benzthiazide,Brinzolamide,Chlorothiazide,Cyclothiazide,Diclofenamide,Dorzolamide,Ellagic Acid,Ethoxzolamide,Hydrochlorothiazide,Hydroflumethiazide,Methazolamide,Methyclothiazide,Topiramate,Trichlormethiazide,Zonisamide,Brinzolamide
CA5A	Ellagic Acid,Zonisamide,Acetazolamide
CA7	Diclofenamide,Ellagic Acid,Ethoxzolamide,Methazolamide,Zonisamide,Cyclosporine
CAMLG	Cyclosporine
CASR	Cinacalcet
CAT	Fomepizole
CEACAM5	2,2,5,5-TETRAMETHYL-3-(SULFANYLMETHYL)-2,5-DIHYDRO-1H-PYRROL-1-OL
CELA2A	2-(2-HYDROXY-CYCLOPENTYL)-PENT-4-ENAL
CES1	(1S,7S,8S,8AR)-1,2,3,7,8,8A-HEXAHYDRO-7-METHYL-8-[2-[(2R,4R)-TETRAHYDRO-4-HY DROXY-6-OXO-2H-PYRAN-2-YL]ETHYL]-1-NAPHTHALENOL,L-Carnitine,Oseltamivir,Creatine
CKB	Creatine
CRH	Corticotropin
CRYZ	Dicoumarol
CTH	Pyridoxal Phosphate,Aminoglutethimide
CYP11A1	Aminoglutethimide
CYP1A2	lidocaine patch,Paliperidone
CYP3A4	pradefovir mesylate,NADH
CYP4A11	NADH
DAO	(2E)-3-(3,4-DIHYDROXYPHENYL)-2-IMINOPROPANOIC ACID
DAPK1	6-(3-AMINOPROPYL)-4,9-DIMETHYLPYRROLO[3,4-C]CARBAZOLE-1,3(2H,6H)-DIONE
DHCR7	NADH
DRD4	Apomorphine,Aripiprazole,Asenapine,Bromocriptine,Cabergoline,Chlorpromazine,Clozapine,Dopamine,L-DOPA,Lisuride,Loxapine,Methotrimeprazine,Olanzapine,Paliperidone,Pergolide,Pramipexole,Promazine,Propiomazine,Quetiapine,Remoxipride,Risperidone,Ropinirole,Rotigotine,SLV 308,Thiethylperazine,Ziprasidone,Amifostine
ENPP1	Ribavirin,ING-1
EPCAM	oportuzumab monatox,5-[(2-methyl-5-{[3-(trifluoromethyl)phenyl]carbamoyl}phenyl)amino]pyridine-3-carboxamide
EPHA7	5-[(2-methyl-5-{[3-(trifluoromethyl)phenyl]carbamoyl}phenyl)amino]pyridine-3-carboxamide
FAAH	4-(3-{[5-(trifluoromethyl)pyridin-2-yl]oxy}benzyl)piperidine-1-carboxylic acid,4-(quinolin-3-ylmethyl)piperidine-1-carboxylic acid,Thiopental,2,5-DICHLORO-N-(5-CHLORO-1,3-BENZOXAZOL-2-YL)BENZENESULFONAMIDE
FBP1	2,5-DICHLORO-N-[5-METHOXY-7-(6-METHOXYPYRIDIN-3-YL)-1,3-BENZOXAZOL-2-YL]BENZENESULFONAMIDE,4-AMINO-N-[(2-SULFANYLETHYL)CARBAMOYL]BENZENESULFONAMIDE,Adenosine monophosphate,N-[7-(3-AMINOPHENYL)-5-METHOXY-1,3-BENZOXAZOL-2-YL]-2,5-DICHLOROBENZENESULFONAMIDE,5-AMINO-NAPHTALENE-2-MONOSULFONATE
FGF1	Amlexanox,Pazopanib,Pentosan Polysulfate,(2S)-2-{[HYDROXY(4-IODOBENZYL)PHOSPHORYL]METHYL}PENTANEDIOIC ACID
FOLH1	Capromab,L-Glutamic Acid,N-({(1R)-1-carboxy-2-[(4-fluorobenzyl)sulfanyl]ethyl}carbamoyl)-L-glutamic acid,Cyclothiazide
FXYD2	Cyclothiazide
GABRA1	Adinazolam,Alprazolam,Amobarbital,Amoxapine,Aprobarbital,Barbital,Bromazepam,Butalbital,Butethal,Clobazam,Clorazepate,Clotiazepam,Desflurane,Diazepam,Enflurane,Ergoloid mesylate,Estazolam,Estazolam,Eszopiclone,Ethanol,Ethchlorvynol,Etomidate,Fludiazepam,Flumazenil,Flumazenil,Flurazepam,Ginkgo biloba,Glutethimide,Glutethimide,Halazepam,Halothane,Hexobarbital,Isoflurane,Lorazepam,Meprobamate,Metharbital,Metharbital,Methoxyflurane,Methoxyflurane,Methylphenobarbital,Methyprylon,Olanzapine,Oxazepam,Pentobarbital,Phenobarbital,Picrotoxin,Primidone,Primidone,Propofol,Secobarbital,Sevoflurane,Talbutal,Talbutal,Temazepam,Temazepam,Thiamylal,Topiramate,Topiramate,Triazolam,Zaleplon,Zolpidem,Zopiclone
GABRA3	Adinazolam,Amobarbital,Aprobarbital,Barbital,Barbituric acid derivative,Bromazepam,Butalbital,Butethal,Clotiazepam,Diazepam,Estazolam,Eszopiclone,Fludiazepam,Flunitrazepam,Flurazepam,Halazepam,Heptabarbital,Hexobarbital,Meprobamate,Metharbital,Methylphenobarbital,Midazolam,Oxazepam,Pentobarbital,Primidone,Secobarbital,Talbutal,Temazepam,Thiopental,Triazolam,Zolpidem,Zopiclone
GABRA4	Amobarbital,Aprobarbital,Barbital,Barbituric acid derivative,Bromazepam,Butalbital,Butethal,Flunitrazepam,Flurazepam,Heptabarbital,Hexobarbital,Meprobamate,Metharbital,Methylphenobarbital,Midazolam,Oxazepam,Pentobarbital,Primidone,Secobarbital,Talbutal,Temazepam,Thiopental,Triazolam
GABRA5	Amobarbital,Barbital,Barbituric acid derivative,Bromazepam,Butalbital,Butethal,Clotiazepam,Diazepam,Estazolam,Eszopiclone,Fludiazepam,Flumazenil,Flunitrazepam,Flurazepam,Halazepam,Heptabarbital,Hexobarbital,Meprobamate,Metharbital,Methylphenobarbital,Midazolam,Oxazepam,Pentobarbital,Primidone,Secobarbital,Talbutal,Temazepam,Thiopental,Triazolam,Zopiclone
GABRB1	Adinazolam,Bromazepam,Clotiazepam,Diazepam,Estazolam,Fludiazepam,Flurazepam,Gamma Hydroxybutyric Acid,Halazepam,Lindane,Midazolam,Oxazepam,Temazepam,Triazolam
GABRB2	Adinazolam,Bromazepam,Clotiazepam,Diazepam,Estazolam,Fludiazepam,Flurazepam,Fospropofol,Ginkgo biloba,Halazepam,Midazolam,Oxazepam,Propofol,Temazepam,Triazolam
GABRG1	Adinazolam,Bromazepam,Clotiazepam,Diazepam,Estazolam,Fludiazepam,Flurazepam,Halazepam,Midazolam,Oxazepam,Temazepam,Triazolam
GABRG2	Adinazolam,Bromazepam,Clotiazepam,Diazepam,Estazolam,Fludiazepam,Flumazenil,Flurazepam,Ginkgo biloba,Halazepam,Midazolam,Oxazepam,Temazepam,Triazolam
GAMT	Creatine,Guanidine
GATM	Glycine,L-Ornithine
GGCX	Anisindione,Coagulation Factor IX,Coagulation factor VIIa,Drotrecogin alfa,L-Glutamic Acid,Menadione,Phylloquinone
GGT1	Glutathione
GLYAT	Glycine
GLYATL1	Glycine
GNMT	Glycine,S-Adenosylmethionine
GOT1	L-Aspartic Acid,L-Cysteine,L-Glutamic Acid,Pyridoxal Phosphate
GPX2	Glutathione
GRM1	L-Glutamic Acid
GRM4	L-Glutamic Acid
GSTA1	Glutathione
GSTA2	Chloroquine,Glutathione
GSTM3	Glutathione
HAGH	Glutathione
HIBADH	NADH
HPGD	NADH
HSD11B1	(1S,3R,4S,5S,7S)-4-{[2-(4-METHOXYPHENOXY)-2-METHYLPROPANOYL]AMINO}ADAMANTANE-1-CARBOXAMIDE,(2R)-1-[(4-tert-butylphenyl)sulfonyl]-2-methyl-4-(4-nitrophenyl)piperazine,(3,3-dimethylpiperidin-1-yl)(6-(3-fluoro-4-methylphenyl)pyridin-2-yl)methanone,(5R)-2-[(2-fluorophenyl)amino]-5-(1-methylethyl)-1,3-thiazol-4(5H)-one,(5S)-2-(cyclooctylamino)-5-methyl-5-propyl-1,3-thiazol-4(5H)-one,(5S)-2-{[(1S)-1-(2-fluorophenyl)ethyl]amino}-5-methyl-5-(trifluoromethyl)-1,3-thiazol-4(5H)-one,(5S)-2-{[(1S)-1-(4-fluorophenyl)ethyl]amino}-5-(1-hydroxy-1-methylethyl)-5-methyl-1,3-thiazol-4(5H)-one,1-{[(3R)-3-methyl-4-({4-[(1S)-2,2,2-trifluoro-1-hydroxy-1-methylethyl]phenyl}sulfonyl)piperazin-1-yl]methyl}cyclopropanecarboxamide,2-(2-CHLORO-4-FLUOROPHENOXY)-2-METHYL-N-[(1R,2S,3S,5S,7S)-5-(METHYLSULFONYL)-2-ADAMANTYL]PROPANAMIDE,2-(6-{[(3-chloro-2-methylphenyl)sulfonyl]amino}pyridin-2-yl)-N,N-diethylacetamide,N-{1-[(1-carbamoylcyclopropyl)methyl]piperidin-4-yl}-N-cyclopropyl-4-[(1S)-2,2,2-trifluoro-1-hydroxy-1-methylethyl]benzamide,N-cyclopropyl-N-(trans-4-pyridin-3-ylcyclohexyl)-4-[(1S)-2,2,2-trifluoro-1-hydroxy-1-methylethyl]benzamide,NADH,Prednisone
HSD17B6	Succinic acid
HTR1B	Almotriptan,Amitriptyline,Amoxapine,Apomorphine,Aripiprazole,Asenapine,Bopindolol,Bromocriptine,Cabergoline,Clozapine,Dihydroergotamine,Eletriptan,Ergotamine,Frovatriptan,Lisuride,Loxapine,MAP-0004,Methysergide,Naratriptan,NXN-188,Olanzapine,Ondansetron,Penbutolol,Pergolide,Pindolol,Pramipexole,Propranolol,Quetiapine,Rizatriptan,Ropinirole,Sumatriptan,Yohimbine,Ziprasidone,Zolmitriptan
HTR2A	3,4-Methylenedioxymethamphetamine,ACP-103,Amisulpride,Amitriptyline,Amoxapine,Amperozide,Apomorphine,Aripiprazole,Asenapine,BL-1020,Bromocriptine,Cabergoline,Chlorpromazine,Chlorprothixene,Cinitapride,Cisapride,Clomipramine,Clozapine,Cyclobenzaprine,Cyproheptadine,Desipramine,Dimethyltryptamine,Donepezil,Doxepin,Epicept NP-1,Epinastine,Ergotamine,Flupentixol,Haloperidol,Imipramine,Ketamine,Lisuride,Loxapine,Lurasidone,MAP-0004,Maprotiline,Mesoridazine,Methotrimeprazine,Methysergide,Mianserin,Minaprine,Mirtazapine,MMDA,Nefazodone,Nortriptyline,Olanzapine,Paliperidone,Paroxetine,Pergolide,Pramipexole,Promazine,Promethazine,Propiomazine,Quetiapine,Remoxipride,Risperidone,Ropinirole,Sertindole,Tegaserod,Thioridazine,Trazodone,Trimipramine,Yohimbine,Ziprasidone,Zuclopenthixol acetate,Zuclopenthixol decanoate
IMPA2	Lithium
INS	MYRISTIC ACID
KCNA1	Amitriptyline,Dalfampridine,Desflurane,Enflurane,Isoflurane,Methoxyflurane,Nifedipine,Sevoflurane
KCNA4	Dalfampridine
KCNJ3	Halothane
KCNMA1	Bendroflumethiazide,Chlorzoxazone,Cromoglicic acid,Diazoxide,Halothane,Hydrochlorothiazide,Hydroflumethiazide,Miconazole,Procaine
KLK1	Aprotinin
LCMT1	L-Leucine
LDHB	NADH
LEPRE1	L-Proline,Succinic acid,Vitamin C
LIPT1	Lipoic Acid
MAP1A	Estramustine
MC2R	Corticotropin,Cosyntropin
MMP7	(1R)-N,6-DIHYDROXY-7-METHOXY-2-[(4-METHOXYPHENYL)SULFONYL]-1,2,3,4-TETRAHYDROISOQUINOLINE-1-CARBOXAMIDE,5-METHYL-3-(9-OXO-1,8-DIAZA-TRICYCLO[10.6.1.013,18]NONADECA-12(19),13,15,17-TETRAEN-10-YLCARBAMOYL)-HEXANOIC ACID,Marimastat,N4-HYDROXY-2-ISOBUTYL-N1-(9-OXO-1,8-DIAZA-TRICYCLO[10.6.1.013,18]NONADECA-12(19),13,15,17-TETRAEN-10-YL)-SUCCINAMIDE
MST4	[4-({4-[(5-CYCLOPROPYL-1H-PYRAZOL-3-YL)AMINO]QUINAZOLIN-2-YL}IMINO)CYCLOHEXA-2,5-DIEN-1-YL]ACETONITRILE
NAE1	Adenosine triphosphate
NDUFA8	NADH
NDUFB5	NADH
NOS2	(2S)-2-methyl-2,3-dihydrothieno[2,3-f][1,4]oxazepin-5-amine,(3R)-3-[(1,2,3,4-tetrahydroisoquinolin-7-yloxy)methyl]-2,3-dihydrothieno[2,3-f][1,4]oxazepin-5-amine,(3S)-1-(1,3-BENZODIOXOL-5-YLMETHYL)-3-[4-(1H-IMIDAZOL-1-YL)PHENOXY]PIPERIDINE,1-(6-CYANO-3-PYRIDYLCARBONYL)-5′,8′-DIFLUOROSPIRO[PIPERIDINE-4,2′(1′H)-QUINAZOLINE]-4′-AMINE,1-[4-(AMINOMETHYL)BENZOYL]-5′-FLUORO-1′H-SPIRO[PIPERIDINE-4,2′-QUINAZOLIN]-4′-AMINE,4-({4-[(4-methoxypyridin-2-yl)amino]piperidin-1-yl}carbonyl)benzonitrile,4-(1,3-BENZODIOXOL-5-YLOXY)-2-[4-(1H-IMIDAZOL-1-YL)PHENOXY]-6-METHYLPYRIMIDINE,4-(1,3-BENZODIOXOL-5-YLOXY)-2-[4-(1H-IMIDAZOL-1-YL)PHENOXY]PYRIMIDINE,4-(1H-IMIDAZOL-1-YL)PHENOL,5-(4′-AMINO-1′-ETHYL-5′,8′-DIFLUORO-1′H-SPIRO[PIPERIDINE-4,2′-QUINAZOLINE]-1-YLCARBONYL)PICOLINONITRILE,Dexamethasone,ETHYL 4-[(4-CHLOROPYRIDIN-2-YL)AMINO]PIPERIDINE-1-CARBOXYLATE,ETHYL 4-[(4-METHYLPYRIDIN-2-YL)AMINO]PIPERIDINE-1-CARBOXYLATE,L-Arginine,L-Citrulline,Miconazole,N-[2-(1,3-BENZODIOXOL-5-YL)ETHYL]-1-[2-(1H-IMIDAZOL-1-YL)-6-METHYLPYRIMIDIN-4-YL]-D-PROLINAMIDE,N-[2-(4-AMINO-5,8-DIFLUORO-1,2-DIHYDROQUINAZOLIN-2-YL)ETHYL]-3-FURAMIDE,N-[2-(6-AMINO-4-METHYLPYRIDIN-2-YL)ETHYL]-4-CYANOBENZAMIDE,Triflusal
P2RY12	Clopidogrel,Epoprostenol,Prasugrel,Ticagrelor,Ticlopidine,Treprostinil
PADI2	L-Citrulline
PC	5-(HEXAHYDRO-2-OXO-1H-THIENO[3,4-D]IMIDAZOL-6-YL)PENTANAL,Biotin,Pyruvic acid
PCSK1	Insulin Regular,Insulin, porcine
PDE3A	Aminophylline,Amrinone,Anagrelide,Cilostazol,Ibudilast,Levosimendan,Milrinone,Oxtriphylline,Theophylline,Tofisopam
PDE5A	3-ISOBUTYL-1-METHYLXANTHINE,5-ethoxy-4-(1-methyl-7-oxo-3-propyl-6,7-dihydro-1H-pyrazolo[4,3-d]pyrimidin-5-yl)thiophene-2-sulfonamide,3-ISOBUTYL-1-METHYLXANTHINE,Avanafil,Dipyridamole,OSI-461,Pentoxifylline,Sildenafil,Tadalafil,Theophylline,Udenafil,Vardenafil
PDK2	(N-{4-[(ETHYLANILINO)SULFONYL]-2-METHYLPHENYL}-3,3,3-TRIFLUORO-2-HYDROXY-2-METHYLPROPANAMIDE,4-({(2R,5S)-2,5-DIMETHYL-4-[(2R)-3,3,3-TRIFLUORO-2-HYDROXY-2-METHYLPROPANOYL]PIPERAZIN-1-YL}CARBONYL)BENZONITRILE,N-(2-AMINOETHYL)-2-{3-CHLORO-4-[(4-ISOPROPYLBENZYL)OXY]PHENYL} ACETAMIDE
PDXP	Pyridoxal Phosphate
PHGDH	NADH
PHYH	Antihemophilic Factor,Vitamin C
PIPOX	Glycine
PRKACA	(1S)-1-(1H-INDOL-3-YLMETHYL)-2-(2-PYRIDIN-4-YL-[1,7]NAPHTYRIDIN-5-YLOXY)-EHYLAMINE,(1S)-2-(1H-INDOL-3-YL)-1-[({5-[(E)-2-PYRIDIN-4-YLVINYL]PYRIDIN-3-YL}OXY)METHYL]ETHYLAMINE,(1S)-2-(1H-INDOL-3-YL)-1-{[(5-ISOQUINOLIN-6-YLPYRIDIN-3-YL)OXY]METHYL}ETHYLAMINE,(2R)-2-(4-CHLOROPHENYL)-2-[4-(1H-PYRAZOL-4-YL)PHENYL]ETHANAMINE,(2R)-2-(4-CHLOROPHENYL)-2-PHENYLETHANAMINE,(2S)-1-(1H-INDOL-3-YL)-3-{[5-(3-METHYL-1H-INDAZOL-5-YL)PYRIDIN-3-YL]OXY}PROPAN-2-AMINE,(2S)-1-(6H-INDOL-3-YL)-3-{[5-(7H-PYRAZOLO[3,4-C]PYRIDIN-5-YL)PYRIDIN-3-YL]OXY}PROPAN-2-AMINE,(2S)-1-{[5-(1H-INDAZOL-5-YL)PYRIDIN-3-YL]OXY}-3-[(7AS)-7AH-INDOL-3-YL]PROPAN-2-AMINE,(2S)-1-{[5-(3-METHYL-1H-INDAZOL-5-YL)PYRIDIN-3-YL]OXY}-3-PHENYLPROPAN-2-AMINE,(2S)-2-(4-CHLOROPHENYL)-2-[4-(1H-PYRAZOL-4-YL)PHENYL]ETHANAMINE,(4R,2S)-5′-(4-(4-CHLOROBENZYLOXY)PYRROLIDIN-2-YLMETHANESULFONYL)ISOQUINOLINE,(R)-TRANS-4-(1-AMINOETHYL)-N-(4-PYRIDYL) CYCLOHEXANECARBOXAMIDE,(S)-1-PHENYL-1-[4-(9H-PURIN-6-YL)PHENYL]METHANAMINE,(S)-2-METHYL-1-[(4-METHYL-5-ISOQUINOLINE)SULFONYL]-HOMOPIPERAZINE,1-(5-ISOQUINOLINESULFONYL)-2-METHYLPIPERAZINE,1-[4-(4-chlorobenzyl)-1-(7H-pyrrolo[2,3-d]pyrimidin-4-yl)piperidin-4-yl]methanamine,1-[4-(4-chlorophenyl)-1-(7H-pyrrolo[2,3-d]pyrimidin-4-yl)piperidin-4-yl]methanamine,2-[4-(3-METHYL-1H-PYRAZOL-4-YL)PHENYL]ETHANAMINE,3-(1H-INDOL-3-YL)-4-{1-[2-(1-METHYLPYRROLIDIN-2-YL)ETHYL]-1H-INDOL-3-YL}-1H-PYRROLE-2,5-DIONE,3-pyridin-4-yl-1H-indazole,3-PYRIDIN-4-YL-2,4-DIHYDRO-INDENO[1,2-.C.] PYRAZOLE,4-(4-chlorobenzyl)-1-(7H-pyrrolo[2,3-d]pyrimidin-4-yl)piperidin-4-aminium,4-(4-CHLOROPHENYL)-4-[4-(1H-PYRAZOL-4-YL)PHENYL]PIPERIDINE,5-(1,4-DIAZEPAN-1-SULFONYL)ISOQUINOLINE,5-benzyl-1,3-thiazol-2-amine,6-{4-[4-(4-CHLOROPHENYL)PIPERIDIN-4-YL]PHENYL}-9H-PURINE,Ellagic Acid,ISOQUINOLINE-5-SULFONIC ACID (2-(2-(4-CHLOROBENZYLOXY)ETHYLAMINO)ETHYL)AMIDE,MYRISTIC ACID,N-[(1S)-2-AMINO-1-(2,4-DICHLOROBENZYL)ETHYL]-5-[2-(METHYLAMINO)PYRIMIDIN-4-YL]THIOPHENE-2-CARBOXAMIDE,N-[2-(4-BROMOCINNAMYLAMINO)ETHYL]-5-ISOQUINOLINE SULFONAMIDE,N-[2-(METHYLAMINO)ETHYL]-5-ISOQUINOLINESULFONAMIDE,N-METHYL-1-[4-(9H-PURIN-6-YL)PHENYL]METHANAMINE
PRKCA	Ellagic Acid,Phosphatidylserine,Tamoxifen,Vitamin E
PRODH	L-Proline
PTGIS	(6E)-7-{6-[(1E)-OCT-1-ENYL]-2,3-DIAZABICYCLO[2.2.1]HEPT-2-EN-5-YL}HEPT-6-ENOIC ACID,Epoprostenol,Phenylbutazone
RBP1	Acitretin,Vitamin A
RLBP1	Vitamin A
S100A2	Olopatadine
S100A4	Trifluoperazine
S100B	(Z)-2-[2-(4-methylpiperazin-1-yl)benzyl]diazenecarbothioamide,2-[(5-hex-1-yn-1-ylfuran-2-yl)carbonyl]-N-methylhydrazinecarbothioamide,Calcium,Olopatadine,ONO-2506
SCARB1	Phosphatidylserine
SDHB	Succinic acid,UBIQUINONE-1
SDSL	Pyridoxal Phosphate
SIGMAR1	Amitriptyline,Dextromethorphan,Dimethyltryptamine,Pentazocine,Remoxipride
SLC1A1	L-Aspartic Acid,L-Glutamic Acid
SLC1A2	L-Glutamic Acid
SLC1A5	L-Asparagine
SLC22A11	Probenecid
SLC23A1	Vitamin C
SLC2A2	Streptozocin
SLC6A1	Guvacine,Tiagabine
SLC6A4	3,4-Methylenedioxymethamphetamine,4-Methoxyamphetamine,Amitriptyline,Amoxapine,Amphetamine,Atomoxetine,Chlorphenamine,Citalopram,Clomipramine,Cocaine,CRx-119,Desipramine,Desvenlafaxine,Dexfenfluramine,Dexmethylphenidate,Dextromethorphan,Dopamine,Doxepin,Duloxetine,Ephedra,Escitalopram,Fenfluramine,Fluoxetine,Fluvoxamine,Imipramine,Levomilnacipran,Loxapine,Mazindol,Methamphetamine,Methylphenidate,Mianserin,Minaprine,Mirtazapine,MMDA,Nefazodone,Nortriptyline,OPC-14523,Paroxetine,Pethidine,Phentermine,Protriptyline,Pseudoephedrine,Sertraline,Sibutramine,Tapentadol,Tramadol,Trazodone,Trimipramine,Venlafaxine,Verapamil
SLC7A11	Acetylcysteine,L-Cystine,L-Glutamic Acid,Riluzole,Sulfasalazine
SLC8A1	Alpha-Linolenic Acid,Icosapent
SMPD3	Phosphatidylserine
SOAT1	Ezetimibe,Hesperetin
SRD5A2	Azelaic Acid,Dutasteride,Finasteride
SST	Cysteamine
SULT1E1	Cyclizine
THNSL1	L-Threonine,Pyridoxal Phosphate
TPO	Carbimazole,Dextrothyroxine,Methimazole,Propylthiouracil
TPSAB1	1-(1′-{[3-(methylsulfanyl)-2-benzothiophen-1-yl]carbonyl}spiro[1-benzofuran-3,4′-piperidin]-5-yl)methanamine,1-[1′-(3-phenylacryloyl)spiro[1-benzofuran-3,4′-piperidin]-5-yl]methanamine
TPSB2	(5-(aminomethyl)-2H-spiro[benzofuran-3,4′-piperidine]-1′-yl)(5-(phenylethynyl)furan-2-yl)methanone
UQCRC2	2-NONYL-4-HYDROXYQUINOLINE N-OXIDE,FAMOXADONE,METHYL (2Z)-2-(2-{[6-(2-CYANOPHENOXY)PYRIMIDIN-4-YL]OXY}PHENYL)-3-METHOXYACRYLATE,METHYL (2Z)-3-METHOXY-2-{2-[(E)-2-PHENYLVINYL]PHENYL}ACRYLATE,UBIQUINONE-2

**Table 3:** Genes with higher or lower gene and protein correlation in cancer vs. normal tissues.

Tissue	Cancer	Genes with higher gene and protein correlation in cancer	Genes with lower gene and protein correlation in cancer
Adrenal Gland	ACC	BCL2L1, CCND1, FN1, FOXO3, PARK7, RAB11A, **RAF1**, RPS6, SHC1, STMN1, TSC2, YAP1, YBX1	BID, **EGFR**, NF2, PDK1, **PIK3CA**, SMAD3, STAT5A
Adrenal Gland	PCPG	**BCL2**, BCL2L1, BCL2L11, ERCC1, FOXO3, GAB2, GATA3, IGFBP2, **JUN**, MAP2K1, PTEN, TP53BP1, TSC2, XRCC1, XRCC5	BECN1, ERBB2, MAPK8, NF2, **NFKB1**
Colon	COAD	ARID1A, CLDN7, NOTCH1, **SRC**, STK11, TGM2, TSC2, YAP1, YBX1	**EGFR**, ERCC1, KIT, MAPK8, MAPK9, MSH2, SMAD4, XRCC1
Frontal Cortex	LGG	AKT1, BCL2, BCL2L11, MAPK1, MYC, NF2, PARK7, TP53, YWHAE	**ANXA1**, CAV1, PIK3CA, RPS6KB1
Lung	LUAD	ARID1A, CCND1, CDH1, **ERBB2**, PTEN, YAP1, YBX1	**BCL2**, BID, CASP9, EGFR, KIT, PIK3CA, PRKCA, SRC
Lung	LUSC	ARID1A, BIRC2, CCNB1, CCND1, CDH1, EIF4EBP1, **ERBB2**, IGFBP2, MSH6, NOTCH3, PTEN, YAP1, YBX1	**CASP3**, CASP9, **KIT**, MAPK8, **PIK3CA**, PRKCA, SRC
Ovary	OV	**ANXA1**, BIRC2, CDH1, CLDN7, ERBB3, **ESR1**, GAB2, GSK3A, TGM2	ACACA, BCL2, CDKN1B, EGFR, PDK1, **PGR**, PIK3CA, PRKCA, RB1, RPS6KB1
Pancreas	PAAD	AKT1, ANXA1, BCL2L1, CLDN7, CTNNA1, ERBB3, GSK3A, IGFBP2, MYC, PEA15, PXN, RAB25, SRC, SYK, XRCC5	BECN1, **CASP7**, MRE11A, RAD50, RPS6KB1, SMAD4
Testis	TGCT	AKT1, ANXA1, BAX, CCND1, CDH1, CDH3, CDKN1A, ERBB2, ERBB3, FN1, IGFBP2, KIT, **MAPK14**, PXN, **RAF1**, TSC2, YWHAE	**AR**, ATM, RAD50, RB1, SMAD4
Urinary Bladder	BLCA	CCNB1, **CCND1**, CLDN7, EIF4EBP1, ERBB3, GSK3A, MYC	**KIT**, PIK3CA, **PRKCA**, SMAD4

Genes in bold are known drug targets.

**Table 4:** Drug targets with higher or lower gene and protein correlation in cancer vs. normal tissues, and related drugs.

Tissue	Cancer	Drug targets with higher gene and protein correlation in cancer	Drug targets with lower gene and protein correlation in cancer
Adrenal Gland	ACC	RAF1 (Sorafenib, iCo-007, Regorafenib)	EGFR (Trastuzumab, Gefitinib, Lapatinib, HuMax-EGFr, IMC-11F8, Afatinib) PIK3CA (Caffeine)
Adrenal Gland	PCPG	BCL2 (Ibuprofen, Paclitaxel, Rasagiline) JUN (Arsenic trioxide, LGD-1550)	NFKB1 (Thalidomide, P54, NOX-700)
Colon	COAD	SRC (Bosutinib, Dasatinib, Ponatinib)	EGFR (Trastuzumab, Panitumumab, Afatinib, CI-1033, IMC-11F8)
Frontal Cortex	LGG	NA	ANXA1 (Amcinonide,Hydrocortisone)
Lung	LUAD	ERBB2 (ado-trastuzumab emtansine, Afatinib, Trastuzumab)	BCL2 (Ibuprofen, Docetaxel) EGFR (Vandetanib, Cetuximab) KIT (Regorafenib, Ponatinib, Sorafenib, Sunitinib) SRC (Ponatinib)
Lung	LUSC	ERBB2 (ado-trastuzumab emtansine, Afatinib, Trastuzumab)	CASP3 (IDN-6556) KIT (OSI-930) SRC (Ponatinib)
Ovary	OV	ANXA1 (Amcinonide, Hydrocortisone) ESR1 (Chlorotrianisene, Conjugated Estrogens, Etonogestrel, Desogestrel, Progesterone, Raloxifene, Estrone, Estradiol, Clomifene, Fulvestrant, Norgestimate, Ethinyl Estradiol, Melatonin, Trilostane, Fluoxymesterone, Estramustine, Allylestrenol, RALOXIFENE CORE)	PGR (Levonorgestrel,Progesterone,Mifepristone,Norgestimate)
Pancreas	PAAD	NA	CASP7 (IDN-6556)
Testis	TGCT	MAPK14 (SCIO-469) RAF1 (Sorafenib, iCo-007, Regorafenib)	AR (Flutamide, Oxandrolone, Nilutamide, Ketoconazole, Fluoxymesterone, Methyltestosterone, Nandrolone decanoate, Enzalutamide)
Urinary Bladder	BLCA	CCND1 (Arsenic trioxide)	KIT (Imatinib, Pazopanib) PRKCA (Tamoxifen)
