# Supplementary material for: Cross-tissue Analysis of Gene and Protein Expression in Normal and Cancer Tissues
Source: Sci Rep. 2016 May 4;6:24799. doi: 10.1038/srep24799 (PMC4855174; doi:10.1038/srep24799)
Supplement: Supplementary Information [file srep24799-s5.pdf]

Cross-tissue Analysis of Gene and Protein Expression in Normal and Cancer Tissues

Idit Kosti[1], Nishant Jain[1], Dvir Aran[1], Atul J. Butte[1] and Marina Sirota[1]

[1] Institute for Computational Health Sciences, University of California, San Francisco, California, United States of America.

Supplementary Figure Legends:

Supplementary Figure S1: Number of genes and proteins measured by HPM (purple) and GTEx (blue).

Supplementary Figure S2: GTEx – HPM scatterplots per tissue. Red: high gene expression-high protein expression gene set; orange: high gene expression-low protein expression gene set, blue: low gene expression-high protein expression gene set; green: low gene expression-low protein expression gene set. Grey: linear regression line.

Supplementary Figure S3: GTEx – HPM Spearman correlations across all GTEx samples per tissue.

Supplementary Figure S4: Hierarchical clustering of samples based on [A] gene expression (GTEx) and [B] protein expression (HPM). Clusters with statistical significance (p-value: <0.05) are marked in red.

Supplementary Figure S5: Proportion of genes above 10% in GTEx-HPM distribution per tissue. Red: high gene expression-high protein expression gene set, orange: high gene expression-low protein expression gene set; blue: low gene expression-high protein expression gene set; green: low gene expression-low protein expression gene set.

Supplementary Figure S6: Distribution of drug targets across four gene sets per tissue. Red: high gene expression-high protein expression gene set; orange: high gene expression-low

protein expression gene set; blue; low gene expression-high protein expression gene set; green: low gene expression-low protein expression gene set.

Supplementary Figure S1: Number of Genes and Proteins Measured by HPM and GTEx

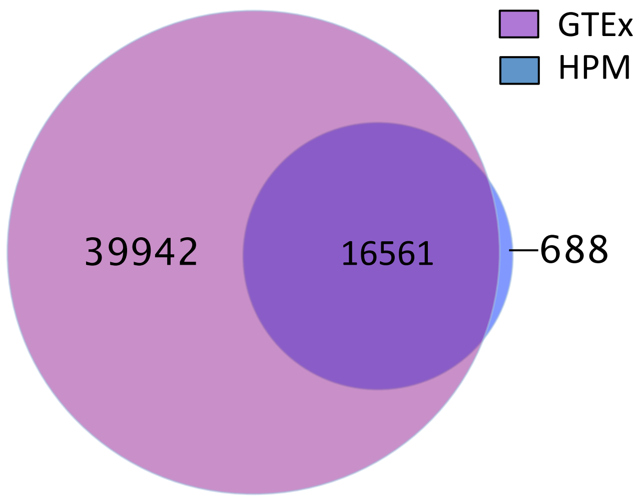

Supplementary Figure S2: GTEx – HPM Expression Scatterplots per Tissue

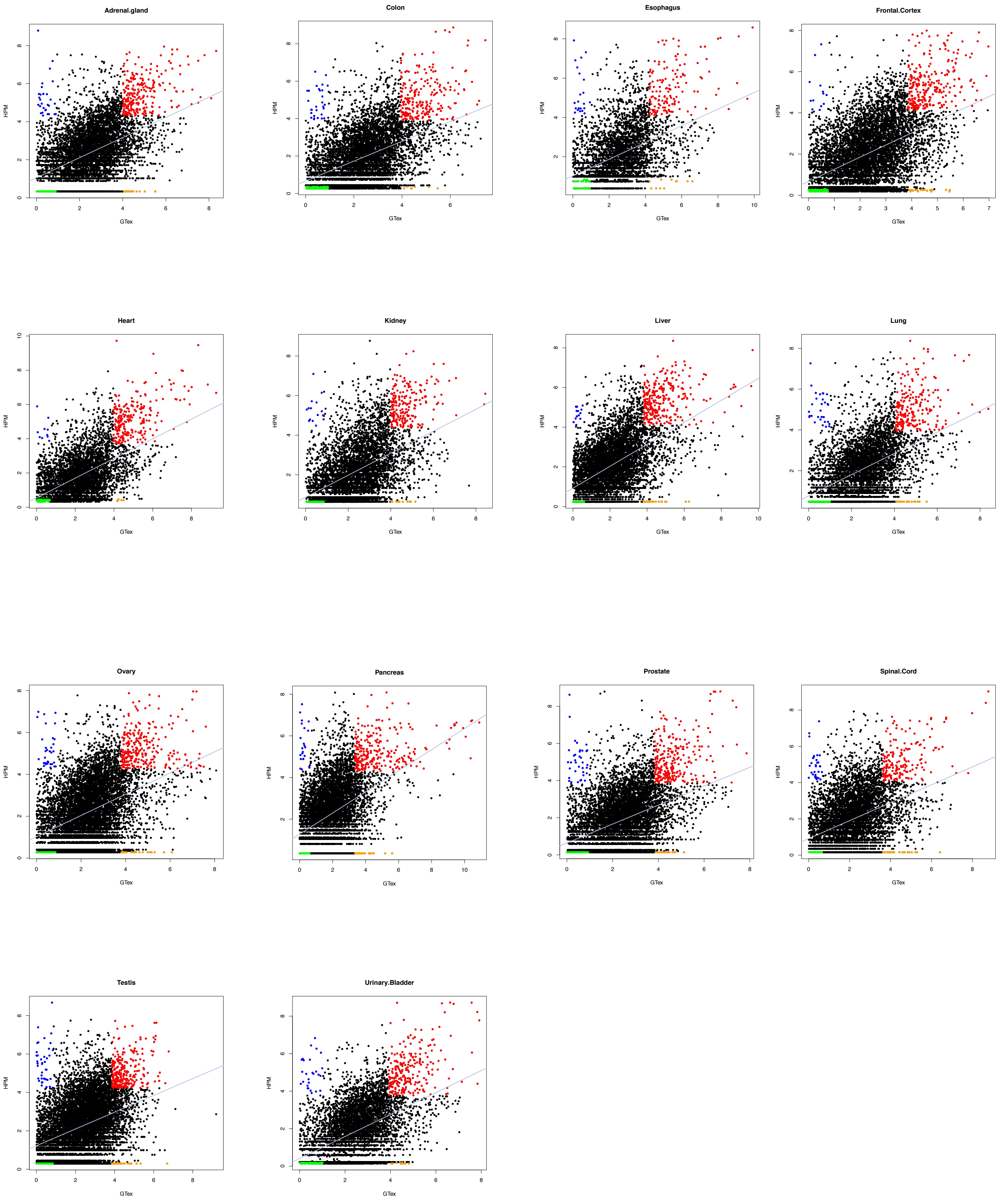

Supplementary Figure S3: GTEx – HPM Spearman Correlations across All GTEx Samples per Tissue

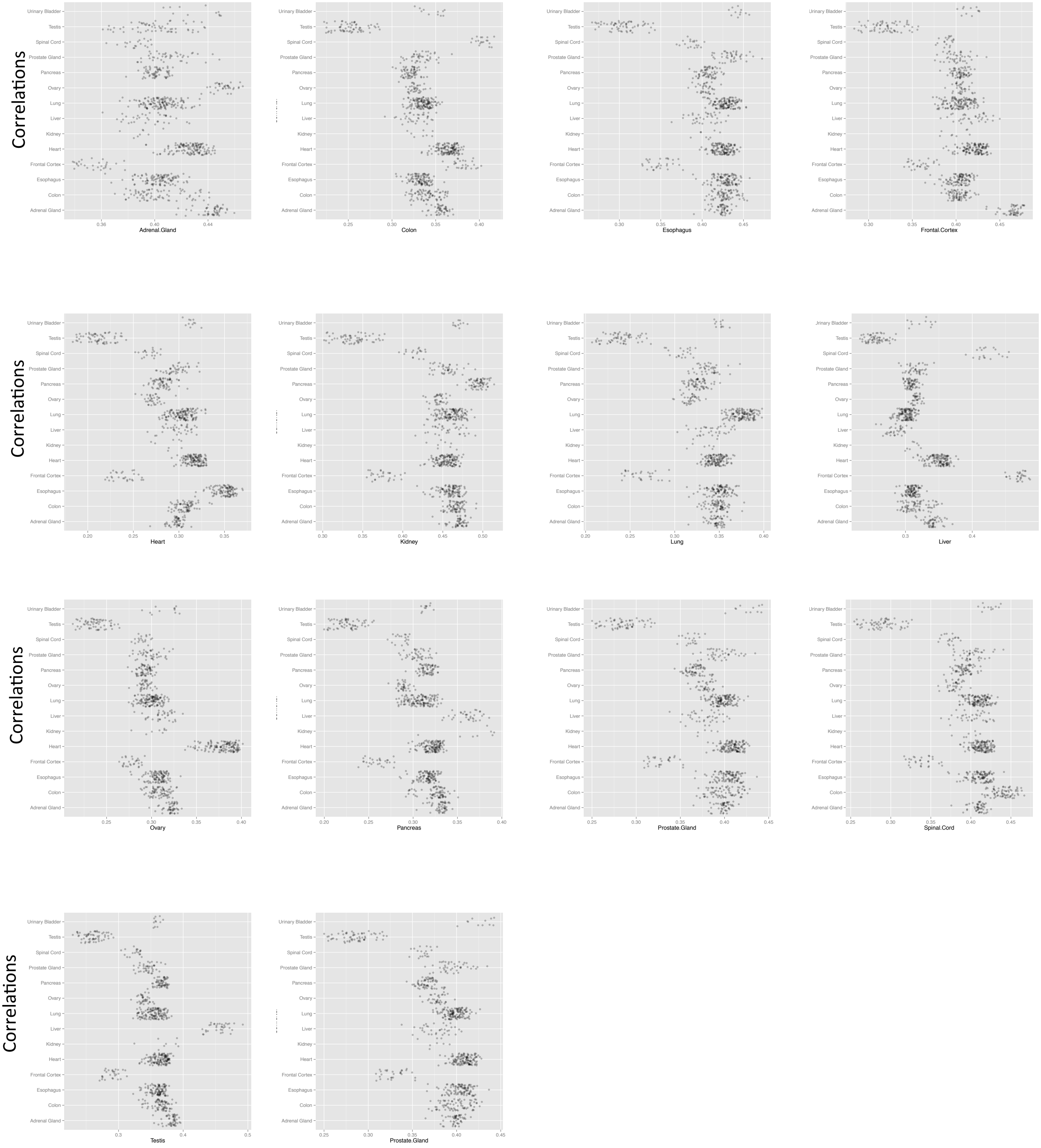

Supplementary Figure S4: Hierarchical Clustering of Samples

A

Hierarchical Clustering of Samples Based on Gene Expression (GTEx)

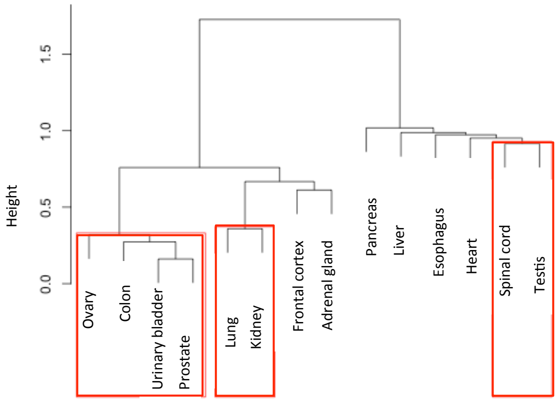

B

Hierarchical Clustering of Samples Based on Protein Expression (HPM)

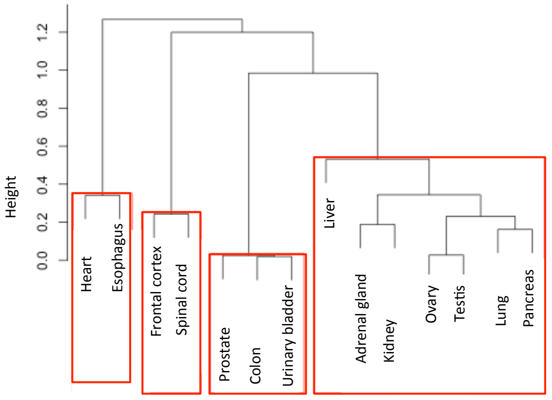

Supplementary Figure S5: Proportion of Corner Case Genes in GTEx-HPM, Distribution per Tissue

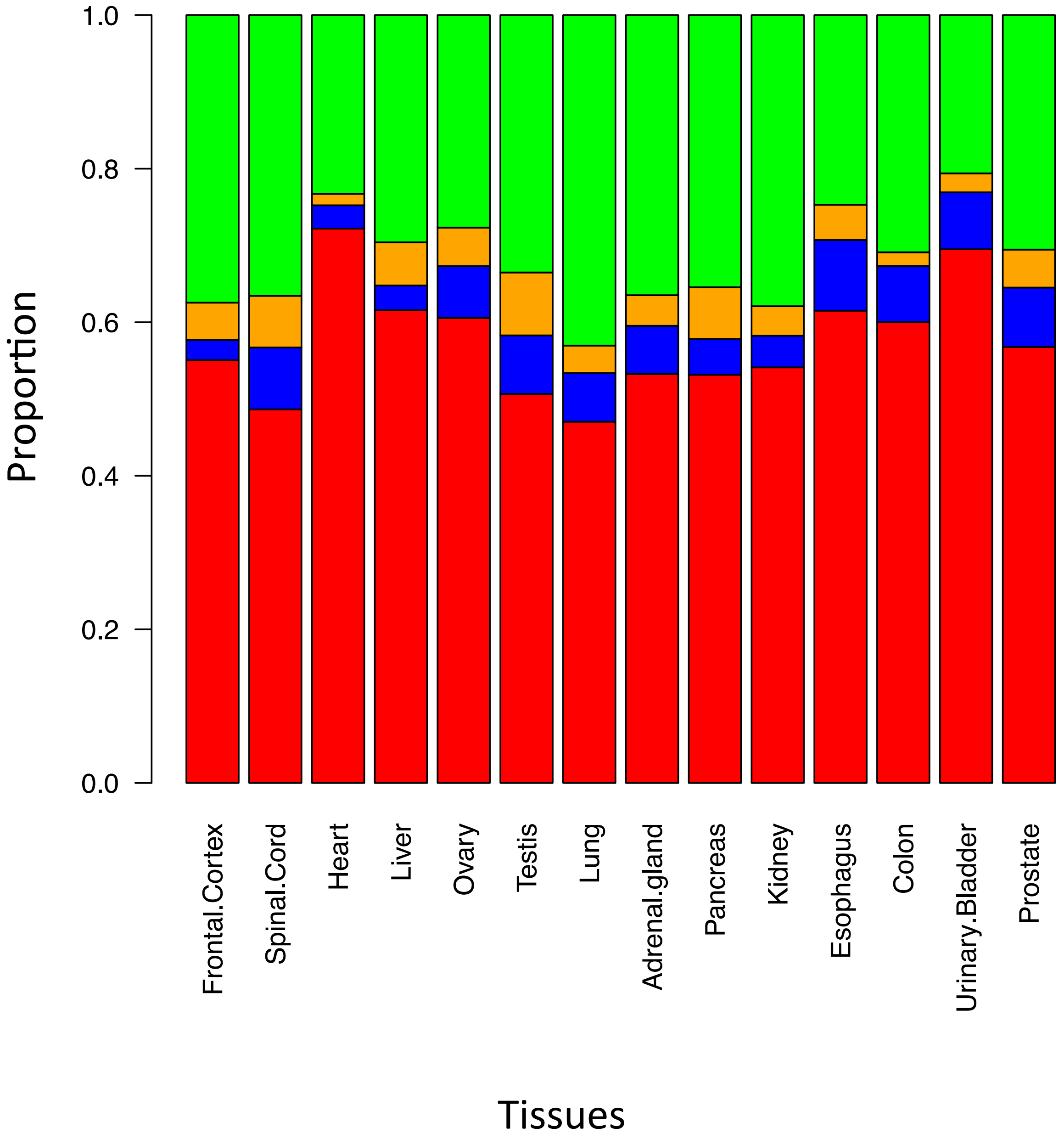

Supplementary Figure S6: Drug Target Distribution across four Corner Case Gene Sets per Tissue

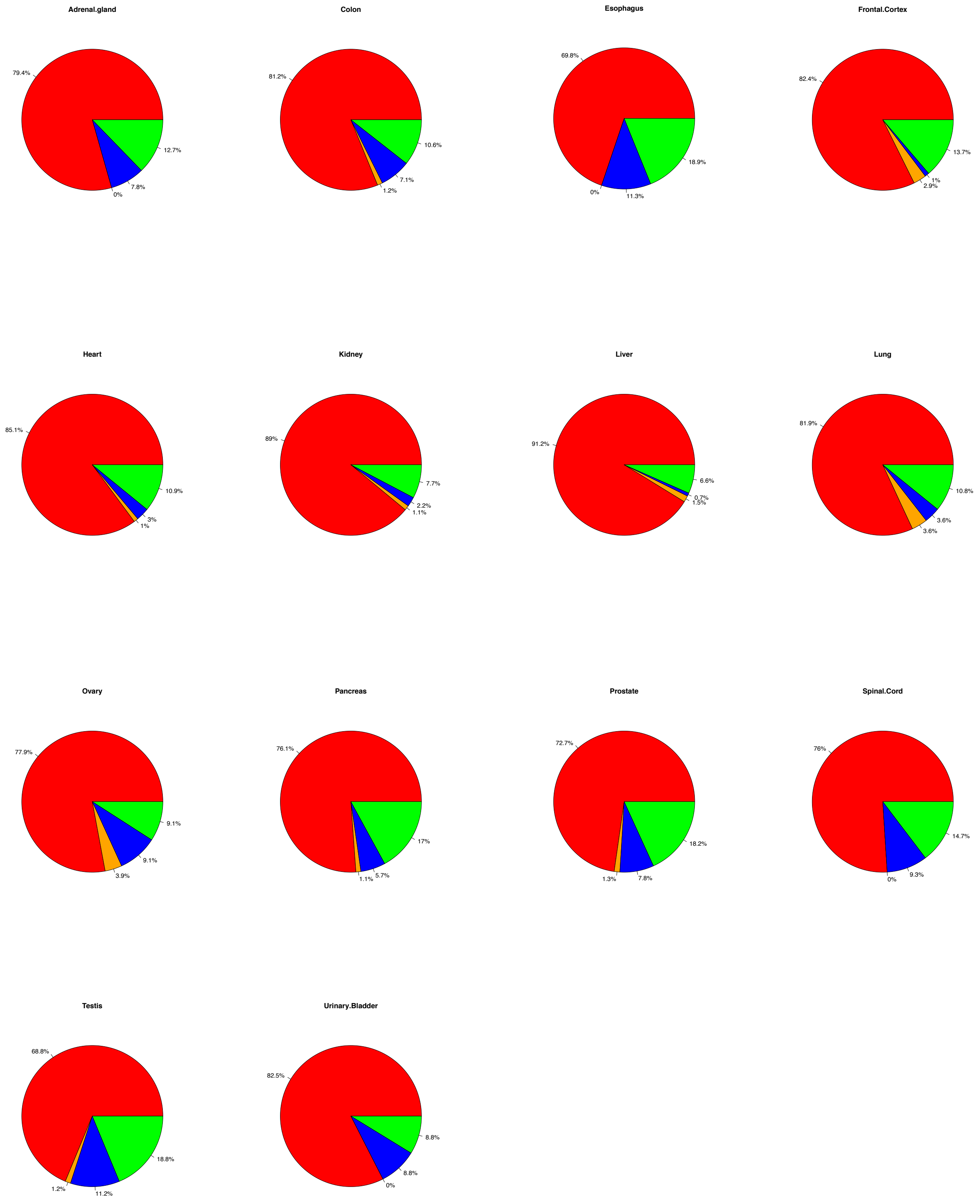

Supplementary Table S1: Genes and proteins expressed per tissue in GTEx and HPM datasets.

Tissue name	Number of genes (GTEx)	Number of genes (HPM)
Frontal cortex	11,340	9,165
Spinal cord	10,737	7,688
Heart	9,937	6,617
Liver	9,738	9,244
Ovary	10,863	10,369
Testis	13,054	11,030
Lung	11,571	6,767
Adrenal gland	10,806	8,591
Pancreas	10,186	9,274
Kidney	10,911	6,803
Esophagus	10,846	5,022
Colon	11,419	8,005
Urinary bladder	11,427	7,697
Prostate	11,661	9,083

Supplementary Table S2: Complete list of genes with statistically significant correlation between RNA expression and protein abundance across in least two tissues.

File: Kosti_TableS2.csv

Supplementary Table S3: Complete list of GO annotations for genes where no RNA expression was observed, but protein expression was observed.

Term	Term name	Count	%	Benjamini p-value
GO:0007600	sensory perception	106	13.22	7.01E-22
GO:0050890	cognition	107	13.34	9.21E-19
GO:0050877	neurological system process	119	14.84	4.41E-15
GO:0007606	sensory perception of chemical stimulus	66	8.23	5.78E-14
GO:0007186	G-protein coupled receptor protein signaling pathway	108	13.47	9.72E-13
GO:0007608	sensory perception of smell	57	7.11	4.26E-11
GO:0004984	olfactory receptor activity	54	6.73	8.28E-09
GO:0006334	nucleosome assembly	21	2.62	9.36E-08
GO:0031497	chromatin assembly	21	2.62	1.60E-07
GO:0065004	protein-DNA complex assembly	21	2.62	3.33E-07
GO:0034728	nucleosome organization	21	2.62	4.50E-07
GO:0007601	visual perception	32	3.99	7.51E-07
GO:0050953	sensory perception of light stimulus	32	3.99	7.51E-07
GO:0006333	chromatin assembly or disassembly	23	2.87	4.15E-06
GO:0009584	detection of visible light	9	1.12	9.32E-06
GO:0007166	cell surface receptor linked signal transduction	127	15.84	1.39E-05
GO:0006323	DNA packaging	21	2.62	1.81E-05
GO:0051606	detection of stimulus	21	2.62	1.96E-05
GO:0009582	detection of abiotic stimulus	15	1.87	6.80E-05
GO:0007156	homophilic cell adhesion	20	2.49	4.09E-04
GO:0009583	detection of light stimulus	11	1.37	4.81E-04
GO:0005198	structural molecule activity	54	6.73	0.002

Supplementary Table S4: Full list of GO annotations for genes where no protein expression was observed, however RNA expression was observed.

Term	Term name	Count	%	Benjamini p-value
GO:0045449	regulation of transcription	281	25.13	2.70E-26
GO:0003677	DNA binding	248	22.18	1.94E-21
GO:0006350	transcription	228	20.39	2.60E-20
GO:0003700	transcription factor activity	131	11.71	1.14E-17
GO:0006355	regulation of transcription, DNA-dependent	192	17.17	2.72E-16
GO:0008270	zinc ion binding	231	20.66	1.96E-16
GO:0051252	regulation of RNA metabolic process	193	17.26	1.05E-15
GO:0030528	transcription regulator activity	170	15.20	5.36E-16
GO:0043565	sequence-specific DNA binding	93	8.32	8.14E-16
GO:0046914	transition metal ion binding	243	21.73	2.49E-10
GO:0007389	pattern specification process	48	4.29	2.82E-09
GO:0003002	regionalization	39	3.48	1.64E-08
GO:0045935	positive regulation of nucleobase, nucleoside, nucleotide and nucleic acid metabolic process	79	7.06	2.28E-08
GO:0045941	positive regulation of transcription	73	6.53	4.41E-08
GO:0048598	embryonic morphogenesis	49	4.38	6.91E-08
GO:0051173	positive regulation of nitrogen compound metabolic process	79	7.06	7.35E-08
GO:0010628	positive regulation of gene expression	73	6.53	1.24E-07
GO:0010557	positive regulation of macromolecule biosynthetic process	77	6.88	6.97E-07
GO:0045893	positive regulation of transcription, DNA-dependent	62	5.54	7.15E-07
GO:0051254	positive regulation of RNA metabolic process	62	5.54	9.10E-07
GO:0001501	skeletal system development	47	4.20	1.41E-06
GO:0031328	positive regulation of cellular biosynthetic process	78	6.97	1.80E-06
GO:0009891	positive regulation of biosynthetic process	78	6.97	3.16E-06
GO:0009952	anterior/posterior pattern formation	28	2.50	3.84E-06
GO:0006357	regulation of transcription from RNA polymerase II promoter	80	7.15	4.32E-06
GO:0010604	positive regulation of macromolecule metabolic process	90	8.05	4.40E-06
GO:0048568	embryonic organ development	30	2.68	2.20E-05
GO:0045944	positive regulation of transcription from RNA polymerase II promoter	48	4.29	3.72E-05
GO:0048562	embryonic organ morphogenesis	24	2.14	2.40E-04
GO:0006955	immune response	71	6.35	2.82E-04

GO:0048736	appendage development	20	1.78	6.41E-04
GO:0060173	limb development	20	1.78	6.41E-04
GO:0043167	ion binding	303	27.10	8.64E-04
GO:0007423	sensory organ development	32	2.865	8.68E-04
GO:0051094	positive regulation of developmental process	36	3.22	1.15E-03
GO:0043169	cation binding	298	26.65	1.16E-03
GO:0035107	appendage morphogenesis	19	1.69	1.23E-03
GO:0035108	limb morphogenesis	19	1.69	1.23E-03
GO:0046872	metal ion binding	295	26.38	1.23E-03
GO:0016564	transcription repressor activity	38	3.39	2.95E-03
GO:0007166	cell surface receptor linked signal transduction	147	13.14	3.63E-03
GO:0051960	regulation of nervous system development	27	2.41	3.94E-03
GO:0045165	cell fate commitment	22	1.96	4.10E-03
GO:0060284	regulation of cell development	28	2.50	4.34E-03
GO:0001654	eye development	21	1.87	5.56E-03
GO:0051216	cartilage development	15	1.34	5.86E-03
GO:0007498	mesoderm development	15	1.34	5.86E-03
GO:0048732	gland development	21	1.87	7.22E-03
GO:0048729	tissue morphogenesis	25	2.23	8.16E-03
GO:0048706	embryonic skeletal system development	15	1.34	8.44E-03
GO:0043370	regulation of CD4-positive, alpha beta T cell differentiation	7	0.62	8.34E-03
GO:0035113	embryonic appendage morphogenesis	16	1.43	8.64E-03
GO:0030326	embryonic limb morphogenesis	16	1.43	8.64E-03
GO:0001657	ureteric bud development	10	0.89	8.43E-03
GO:0045622	regulation of T-helper cell differentiation	6	0.53	8.34E-03
GO:0043010	camera-type eye development	18	1.61	8.32E-03
GO:0045597	positive regulation of cell differentiation	29	2.59	8.78E-03
GO:0045321	leukocyte activation	30	2.68	9.36E-03
GO:0001656	metanephros development	11	0.98	9.73E-03

Supplementary table S5: Complete list of drug targets per tissue for the high gene expression-high protein expression gene set.

File: Kosti_TableS5.csv

Supplementary Table S6: Complete list of drug targets per tissue for the high gene expression-low protein expression gene set.

Tissue	Drug targets
Frontal.Cortex	CA11, CALY, MAPK8IP1
Heart	MAPKAPK2
Liver	CDO1, NFKBIA
Ovary	IGFBP3, JUN, PDK4
Testis	PHOSPHO1
Lung	RAMP3, SCGB1A1, SERPINE1
Pancreas	CLK1
Kidney	VEGFA
Colon	NDUFA1
Prostate	ODC1

Supplementary Table S7: Complete list of drug targets per tissue for the low gene expression-high protein expression gene set.

Tissue	Drug targets
Frontal.Cortex	SERPINA1
Spinal.Cord	ADH1B, AKR1C3, CKMT1A, COL1A1, HSPG2, NID1, TUBA4A
Heart	C4A, C4B, SERPINA1
Liver	ALDH3B1
Ovary	AKR1C1, DPP4, GSTM1, KRT8, PYGM, TF, TUBA4A
Testis	ACTA1, ADH1B, AMBP, ASPH, C4A, C5, CP, FGA, FGG
Lung	ADH1A, CP, MPO
Adrenal.gland	C5, DBH, GPD1, KRT7, ORM1, PON1, SERPINA1, TH
Pancreas	APCS, FGA, GLUD2, HBA1, TUBB2B
Kidney	ACTA1, C5
Esophagus	ACTA1, ADH1C, CP, HBA1, SERPINA1, TUBB2B
Colon	C4A, C4B, CACNA2D1, HBA1, TUBB2B, VCAN
Urinary.Bladder	ADH1A, C4A, C4B, C5, GSTM1, HBA1, SERPINA1
Prostate	ATP2A1, C4A, C4B, PRKCB, TUBB2B, VCAN

Supplementary Table S8: Complete list of drug targets per tissue for the low gene expression-low protein expression gene set.

Tissue	Drug targets
Frontal.Cortex	ABCC1, CYP2D6, FCGR1A, GGT1, HTR1B, ITGA2B, MAN2A1, PAH, PLK1, SEPSECS, SLC25A29, TOP1MT, TRIM13, ZFY
Spinal.Cord	CACNB3, EPHA2, ERBB2, FCGR2C, IKBKG, KCNMA1, KCNN2, MMACHC, MTHFR, PDE7B, TRIM13
Heart	CHM, CTSS, DPP4, EGFR, PAFAH1B3, POLE, PRKCA, PYCR1, TLR2, TLR4, TYMS
Liver	CHM, CHUK, CLCN2, CYP17A1, DHFRL1, JAK2, PIK3CA, PLAT, SLC16A3
Ovary	CUBN, FCGR3A, ITGA2B, KCNMB3, PECR, SEC14L2, SLC7A1
Testis	ATP8A1, AURKB, CACNA1D, CEACAM1, FSHR, GAD1, GLS2, GRM8, KCNMA1, KCNN2, LIPT1, NCAN, NPC1L1, PDE4C, RNASE3
Lung	EGLN3, IL10, INCENP, MAPK10, MGAM, PLK1, PSAT1, SEC14L4, SULT1E1
Adrenal.gland	CACNB3, ESRRG, FCGR1A, IGSF10, ITGB7, NOS3, PDE1B, POLE, PTGS1, PYCR1, SPIRE2, SRD5A1, TPK1
Pancreas	ABCA1, FADS2, FCGR3A, GRIK5, HDAC8, HPD, HSD17B7, INCENP, LPL, MAP3K9, PDE3B, PLA2G4A, SLC13A3, TRIM13, ZFY
Kidney	ADC, EGLN3, HDAC4, HSD11B1, INCENP, PYCR1, S100A12
Esophagus	ABAT, ACY1, CDK6, FGFR4, GCSH, GSTA1, HNMT, LCK, PPAT, ST3GAL5
Colon	ADRBK2, ADSSL1, CA8, CACNA2D2, CHEK1, CYP2D6, ERN1, PDE4B, SPTLC3
Urinary.Bladder	APAF1, CYP2D6, DRD4, PPAT, SPTLC3, TACR1, TBXAS1
Prostate	CPB1, CSNK1G1, F8, FCGR2A, FCGR2C, HSD17B3, INCENP, MAP3K9, PDE1A, PTGIR, SLC12A6, SPTLC3, SRR, TMLHE

Supplementary Table S9: Ranking of highly correlated genes and proteins in cancer vs. normal data per tissue.

File: Kosti_TableS9.csv

Supplementary Table S10: Ranking of highly correlated genes and proteins in cancer vs. normal data per tissue.

File: Kosti_TableS10.csv
